# Predictors for repeated hyperkalemia and potassium trajectories in high-risk patients — A population-based cohort study

**DOI:** 10.1371/journal.pone.0218739

**Published:** 2019-06-21

**Authors:** Kasper Adelborg, Sia Kromann Nicolaisen, Pål Hasvold, Eirini Palaka, Lars Pedersen, Reimar Wernich Thomsen

**Affiliations:** 1 Department of Clinical Epidemiology, Aarhus University Hospital, Aarhus, Denmark; 2 AstraZeneca Nordic, Medical Department, Etterstad, Oslo, Norway; 3 AstraZeneca, Global Payer Evidence, Cambridge, United Kingdom; The Pennsylvania State University, UNITED STATES

## Abstract

Understanding predictors and trajectories of increased potassium may inform testing and treatment of hyperkalemia. We examined predictors for repeated hyperkalemia among patients after first-time renin angiotensin system inhibitor (RASi) prescription, chronic kidney disease (CKD), or chronic heart failure (CHF); and we also examined potassium trajectories in these patients after their first hyperkalemia event. We used Danish population-based registries to identify all patients with first-time RASi prescription, incident CKD, or incident CHF (2000–2012). For patients with a first hyperkalemia event, potassium trajectories over the following 6 months were examined. The predictors associated with repeated hyperkalemia were assessed, with repeated hyperkalemia defined as a potassium test >5.0 mmol/L after the first event within 6 months. Overall 262,375 first-time RASi users, 157,283 incident CKD patients, and 14,600 incident CHF patients were included. Of patients with a first hyperkalemia event, repeated hyperkalemia within 6 months occurred in 37% of RASi users, 40% with CKD, and 49% of patients with CHF. Predictors included severe hyperkalemia, low eGFR, diabetes, and spironolactone use. In all cohorts, the median potassium levels declined over 2–4 weeks after a hyperkalemia event for the first time, but reverted to levels higher than before the initial hyperkalemia event in those who had repeated hyperkalemia. Following hyperkalemia, discontinuation of RASi and spironolactone was common in the RASi and CHF cohorts. Repeated hyperkalemia was common among the explored cohorts. The first hyperkalemia event was an indicator of increased median potassium levels. Predictors may identify patients likely to benefit from intensified monitoring and intervention.

## Introduction

Hyperkalemia, often defined as serum potassium concentration exceeding 5.0 mmol/L is rare in the general population,[1] but occurs in up to 10% of hospitalized patients, depending on patient population, and on the definition of hyperkalemia.[2,3] Patients with chronic kidney disease (CKD),[4] chronic heart failure (CHF),[5] and diabetes[6] are at increased risk for hyperkalemia. Hyperkalemia is triggered by impaired renal potassium excretion due to kidney failure or by pharmacological treatments interfering with potassium homeostasis, such as angiotensin-converting enzyme inhibitors (ACEis), angiotensin II receptor blockers (ARBs) or potassium-sparing diuretics. Patients may also develop hyperkalemia as a result of excessive potassium intake, and/or by compromised ability to shift potassium into the cells caused by *e*.*g*. insulin deficiency.[1]

Hyperkalemia is often considered to be a transient condition, as management strategies restore potassium to normal levels.[3] However, several additional risk factors for hyperkalemia may co-exist in patients, rendering them susceptible to repeated hyperkalemia. More than 40% of patients with CKD, CHF, and diabetes may experience additional hyperkalemia events following an initial event,[4–6] indicating that hyperkalemia may be a continuing issue for these patients.

Little is currently known about the predictors of repeated hyperkalemia events and it is unknown how potassium levels evolve over time in high-risk patients *e*.*g*. patients with CKD or CHF. Further understanding of these trends and trajectories can inform the monitoring strategy for patients at risk and better define the need for ongoing hyperkalemia management. If repeated hyperkalemia events are common, intensified risk factor-guided potassium monitoring may be warranted.

We examined predictors for repeated hyperkalemia events in new-users of renin angiotensin system inhibitors (RASi), and in patients with incident CKD, or incident CHF. We also examined potassium trajectories in these patient cohorts after their first-time hyperkalemia event.

## Materials and methods

### Design, setting, and data sources

We conducted a population-based cohort study in North- and Central Denmark regions from 1 January 2000 to 31 December 2012. This region has a population of 1.8 million people.[7] Denmark has a tax-supported health care system that guarantees access to medical care for all inhabitants. Because all Danish inhabitants are assigned a unique identification number, data from the different registries can be linked at the individual level with virtually complete follow-up. The Danish National Patient Registry (DNPR) is an administrative registry, which collects data on hospital discharge diagnoses from all non-psychiatric hospitals since 1977 and on emergency room visits and hospital outpatient contacts since 1995, coded according to the *International Classification of Diseases*, *Tenth Revision* (ICD-10) since 1994.[8] Laboratory data, including all measurements from hospital inpatient departments, outpatient departments, and offices of general practitioners (GPs) were obtained from the Clinical Laboratory Information System Research Database (LABKA) using Nomenclature for Properties and Units (NPU) codes.[9] The Aarhus University Prescription Database (AUPD) contains data on all prescriptions dispensed in community pharmacies for reimbursed medicines since 1998, recorded according to Anatomical Therapeutic Chemical Classification system (ATC) codes.[10]

### Study populations

The study population consisted of cohorts at particular high risk of hyperkalemia: RASi new-users, patients with CKD, and patients with CHF.[1]

The RASi new-user cohort was defined and followed from a first-ever filled drug prescription for RASi, either ARBs and/or ACEis.

The CKD cohort was defined as patients with a first-time occurrence of one of the following: (1) two creatinine measurements, more than 90 days apart, both with an eGFR <60 mL/min/1.73m^2^; (2) an incident hospitalization with a diagnosis of CKD; (3) or hospital-based codes for renal dialysis.

We used the DNPR to identify all patients with first-time hospital inpatient or outpatient department admission with a primary or secondary discharge diagnosis of heart failure. To increase the positive predictive value of heart failure diagnoses in the DNPR, which is only around 80%,[11] and to ensure inclusion of a CHF population with established disease, we required patients to have echocardiography performed within 12 months before, during or 12 months after index hospitalization admission date. Patients should also have had at least one prescription for RASi and one prescription for beta blocker within 12 months after their hospital admission date, however concomitant use of RASi and beta blockers was not required. Patients were defined as having CHF on the first date of fulfilling all above criteria and were followed from this date. Because patients were allowed to enter more than one cohort, some overlap between the cohorts was expected.

### Predictors of repeated hyperkalemia

All patients were followed for hyperkalemia events defined as potassium levels >5.0 mmol/L, measured during inpatient hospitalization, outpatient departments visits, or at GP visits, after the start of their index condition of RASi initiation, CKD, or CHF. After the first hyperkalemia event, we defined a 6-month trajectory period. During this period, repeated hyperkalemia was defined as a second potassium test >5.0 mmol/L recorded after the first hyperkalemia event (*i*.*e*., ≥2 potassium tests >5.0 mmol/L within 6 months). The remaining patients only had 1 potassium test >5.0 mmol/L (*i*.*e*., the test defining the first hyperkalemia event) recorded within 6 months. We then examined predictors of repeated hyperkalemia, including sex, severity of the first hyperkalemia event, and eGFR level (and log eGFR) as recorded in the LABKA registry. Information on history of comorbidities were obtained using hospital in- and outpatient diagnoses, primary and secondary diagnoses from 1994 until date of first recorded hyperkalemia, including diabetes, CKD, heart failure, ischemic heart disease, and other comorbidities. Data were also retrieved on medications potentially interfering with potassium homeostasis: ACEis, ARBs, spironolactone, macrolides, and other drugs, within 1 year prior to the hyperkalemia date. All codes used in the study are provided in S1 Table.

### Potassium trajectories and treatment changes after first-time hyperkalemia

For all patients who experienced first-time hyperkalemia after the start of their index condition (initiation of RASi, CKD or CHF), we assessed all available potassium measurements in the 6 months before and 6 months after the first hyperkalemia event. To understand the interventions potentially associated with changes in potassium levels over time, these trends were described among the patient cohorts receiving in-hospital care, dialysis, and selected drug therapies during the 6 months trajectory periods.

### Statistical analyses

The distribution of the covariates for the patient cohorts was tabulated, and we calculated the time from study inclusion to the first-time hyperkalemia event. Among patients with first-time hyperkalemia, we compared the prevalence of the predictors for patients with more than one hyperkalemia event (repeated) with those who only had one hyperkalemia event. We then calculated prevalence ratios (PRs) using Poisson regression with robust error variance, adjusting for age and sex. To visualize the potassium trajectories, we calculated the median potassium level of the highest weekly potassium measurement per individual. To assist the clinical interpretation and relevance of the results and ensure they reflect the actual measurements in routine clinical practice, the results of all potassium measurements of 50 randomly selected patients were also graphically plotted. Additionally, for each day, we calculated the proportion of potassium tests >5.0 mmol/L divided by the total number of potassium tests at the same day. To explore interventions potentially associated with potassium changes over time, for each day, the proportion of patients (prevalence proportion) dispensing selected prescription drugs of interest was estimated by finding the number of unique persons that had redeemed a prescription that covered this day, divided by all persons alive.[12] We defined the duration in days of each single prescription as the redeemed number of pills in the package purchased at the pharmacy. Similarly, for each day, we estimated the proportion of patients hospitalized and undergoing dialysis, by identifying the proportion of all persons who had an acute inpatient hospitalization or dialysis.

All analyses were performed separately for each of the three patient cohorts.

### Sensitivity analyses

First, we repeated the analyses to focus only on potassium measurements taken at GP offices as the treatment and clinical course of hyperkalemia may differ from that of hospitalized patients. Second, because the mortality among the three patient cohorts was high and may be related to fluctuations in potassium levels, we repeated the analyses excluding patients who died within the 6 month trajectory period. Third, we tested whether the predictors changed if two hyperkalemia events were required to be separated by at least one or two potassium test results below 5.0 mmol/L (defined as recurrent hyperkalemia), and if we required patients to have at least two potassium tests during the trajectory period, representing patients who had regular interaction with the health care system.

All analyses were performed using SAS version 9.4 (SAS Institute Inc).

### Ethics

In Denmark, registry-based research does not require approval from an ethics committee or informed consent from patients. The study was approved by the Danish Data Protection Agency (record number: 2014-54-0922/KEA-2016-15).

## Results

The population comprised of 262,375 new-users of RASi, 157,283 CKD patients, and 14,600 patients with CHF (S2 Table). RASi users were 10 years younger and had a lower prevalence of comorbidities than patients with CKD and CHF. The proportion of women was lower among RASi new-users (50%) than in the CKD cohort (59%), but higher than in the CHF cohort (37%). Many patients were included in more than one cohort, although they were included at different time points (S1 Fig and S3 Table). The majority of the RASi new-users (69%) were not enrolled in the CKD or CHF cohorts. In the CKD cohort, 50% of the patients were also represented in the RASi and/or CHF cohort. As RASi use was used in the disease definition of CHF, only a minority (5%) of patients with CHF were not included in the two other cohorts.

### Predictors of repeated hyperkalemia

Among 262,375 new-users of RASi, 41,818 (16%) had a subsequent first hyperkalemia event, while in 157,283 patients with incident CKD, 28% (n = 43,845) had a first hyperkalemia event, and in 14,600 patients with CHF, 39% (n = 5,634) had a first hyperkalemia event. The median times (25^th^-75^th^ percentile) from study inclusion to first-time hyperkalemia were 2.2 (0.7–4.6) years for RASi new-users, 1.2 (0.09–3.5) years for patients with incident CKD, and 0.6 (0.12–2.1) years for patients with incident CHF.

Of those with a first hyperkalemia event, repeated hyperkalemia within 6 months occurred in 15,654 (37%) of the RASi new-users, in 17,588 (40%) of patients with CKD, and in 2,749 (49%) of patients with CHF.

The impact of patient characteristics, laboratory results, comorbidity, and various drug treatments on the risk of repeated hyperkalemia is shown in Table 1. Predictors appeared relatively comparable across the cohorts (Tables 1 and S4). The severity of potassium increase at first hyperkalemia event was associated with the risk of subsequent repeated hyperkalemia; specifically the PRs increased 2 to 3-fold for initial potassium levels higher than 7 mmol/l, 1.8 to 2.4-fold for potassium levels between 6.1–7.0 mmol/L, and 1.4–1.8-fold for potassium levels between 5.6–6 mmol/L. Other predictors included low eGFR levels, log eGFR, diabetes, and spironolactone use. In the three cohorts, the PRs were generally higher for CHF, CKD, diabetes, and spironolactone use than for ACEis and ARBs.

**Table 1:** Prevalence ratios of clinical predictors in patients with one versus more than one hyperkalemia events during a 6-month trajectory period.

	N = 41,818 RASi new-users			N = 43,845 patients chronic kidney disease			N = 5,634 patients with chronic heart failure		
	1 HK event, n (%)	≥2 HK events, n (%)	PR^a^ (95% CI)	1 HK event, n (%)	≥2 HK events, n (%)	PR^a^ (95% CI)	1 HK event, n (%)	≥2 HK events, n (%)	PR^a^ (95% CI)
**Total**	26,164 (100)	15,654 (100)	N/A	26,257 (100)	17,588 (100)	N/A	2,885 (100)	2,749 (100)	N/A
**Median (range) potassium tests 6 months before**	2.0 (1.0–5.0)	4.0 (1.0–9.0)	N/A	2.00 (1.0–6.0)	4.0 (1.0–9.0)	N/A	5.0 (1.0–11.0)	7.0 (3.0–14.0)	N/A
**Median (range) potassium tests 6 months after**	3.0 (2.0–6.0)	9.0 (4.0–17.0)	N/A	3.0 (2.0–6.0)	9.0 (5.0–18.0)	N/A	4.0 (2.0–8.0)	10.00 (6.0–19.0)	N/A
**Median (range) age**	72.6 (62.8–81.1)	74.9 (65.7–82.0)	N/A	76.7 (67.5–83.8)	75.9 (66.7–82.8)	N/A	75.2 (67.2–82.2)	76.2 (68.5–82.4)	N/A
**Female**	12,304 (47.0)	7,093 (45.3)	0.94 (0.92–0.96)	14,209 (54.1)	8,145 (46.3)	0.86 (0.85–0.88)	1,085 (37.6)	1,055 (38.4)	1.01 (0.94–1.08)
**First-time K+ level (mmol/L)**									
>5.0–5.5	22,759 (87.0)	11,613 (74.2)	0.85 (0.85–0.86)	22,147 (84.3)	12,537 (71.3)	0.85 (0.84–0.85)	2,406 (83.4)	2,014 (73.3)	0.88 (0.85–0.90)
5.6–6.0	2,467 (9.4)	2,605 (16.6)	1.75 (1.66–1.85)	2,884 (11.0)	3,133 (17.8)	1.63 (1.55–1.70)	343 (11.9)	471 (17.1)	1.44 (1.26–1.63)
6.1–6.5	548 (2.1)	810 (5.2)	2.44 (2.20–2.72)	683 (2.6)	1,062 (6.0)	2.30 (2.10–2.53)	72 (2.5)	145 (5.3)	2.12 (1.60–2.79)
6.6–7.0	223 (0.9)	326 (2.1)	2.40 (2.02–2.85)	295 (1.1)	448 (2.5)	2.25 (1.95–2.61)	34 (1.2)	58 (2.1)	1.80 (1.18–2.74)
>7.0	167 (0.6)	300 (1.9)	3.00 (2.48–3.62)	248 (0.9)	408 (2.3)	2.48 (2.12–2.90)	30 (1.0)	61 (2.2)	2.14 (1.38–3.30)
**eGFR groups (mL/min/1.73m2)**									
Not measured	487 (1.9)	261 (1.7)	0.90 (0.77–1.04)	72 (0.3)	141 (0.8)	2.67 (2.01–3.55)	241 (8.4)	164 (6.0)	0.72 (0.59–0.87)
≥60	8,104 (31.0)	2,663 (17.0)	0.57 (0.55–0.60)	180 (0.7)	197 (1.1)	1.32 (1.07–1.62)	381 (13.2)	196 (7.1)	0.55 (0.47–0.64)
45–59	7,035 (26.9)	3,374 (21.6)	0.80 (0.77–0.83)	11,343 (43.2)	5,148 (29.3)	0.67 (0.65–0.69)	596 (20.7)	425 (15.5)	0.76 (0.67–0.84)
30–44	6,068 (23.2)	4,339 (27.7)	1.15 (1.11–1.18)	8,674 (33.0)	5,856 (33.3)	1.02 (1.00–1.05)	825 (28.6)	771 (28.0)	0.97 (0.90–1.06)
15–29	3,484 (13.3)	3,439 (22.0)	1.58 (1.52–1.65)	4,677 (17.8)	4,317 (24.5)	1.40 (1.35–1.46)	613 (21.2)	820 (29.8)	1.38 (1.26–1.51)
<15	821 (3.1)	1,131 (7.2)	2.32 (2.12–2.53)	1,137 (4.3)	1,597 (9.1)	2.04 (1.90–2.20)	181 (6.3)	248 (9.0)	1.43 (1.19–1.71)
Dialysis	165 (0.6)	447 (2.9)	4.78 (4.01–5.70)	174 (0.7)	332 (1.9)	2.54 (2.11–3.05)	48 (1.7)	125 (4.5)	2.77 (2.00–3.84)
**Comorbidities**									
Diabetes	7,799 (29.8)	5,258 (33.6)	1.14 (1.11–1.18)	6,128 (23.3)	4,995 (28.4)	1.19 (1.15–1.23)	883 (30.6)	990 (36.0)	1.18 (1.10–1.27)
CKD	13,519 (51.7)	10,049 (64.2)	1.21 (1.19–1.23)	N/A	N/A	N/A	1,892 (65.6)	2,094 (76.2)	1.15 (1.11–1.19)
Heart failure	4,501 (17.2)	3,785 (24.2)	1.34 (1.29–1.40)	4,628 (17.6)	4,009 (22.8)	1.29 (1.24–1.34)	N/A	N/A	N/A
IHD	6,947 (26.6)	4,683 (29.9)	1.08 (1.05–1.12)	17,945 (68.3)	12,933 (73.5)	1.08 (1.07–1.09)	1,876 (65.0)	1,744 (63.4)	0.97 (0.94–1.01)
**Comedication**									
No RASi	2,292 (8.8)	1,241 (7.9)	0.90 (0.84–0.96)	13,596 (51.8)	8,164 (46.4)	0.90 (0.88–0.92)	376 (13.0)	311 (11.3)	0.87 (0.75–1.00)
Any RASi	23,872 (91.2)	14,413 (92.1)	1.01 (1.00–1.02)	12,661 (48.2)	9,424 (53.6)	1.11 (1.09–1.13)	2,509 (87.0)	2,438 (88.7)	1.02 (1.00–1.04)
ACEis	17,404 (66.5)	10,714 (68.4)	1.03 (1.02–1.04)	9,012 (34.3)	6,869 (39.1)	1.12 (1.10–1.15)	2,102 (72.9)	2,068 (75.2)	1.03 (1.00–1.07)
ARBs	7,958 (30.4)	4,712 (30.1)	1.00 (0.97–1.03)	4,523 (17.2)	3,347 (19.0)	1.11 (1.07–1.16)	664 (23.0)	667 (24.3)	1.05 (0.96–1.16)
Spironolactone	4,334 (16.6)	3,795 (24.2)	1.43 (1.38–1.49)	4,371 (16.6)	3,870 (22.0)	1.34 (1.29–1.39)	1,332 (46.2)	1,408 (51.2)	1.11 (1.05–1.17)
Macrolides	2,765 (10.6)	1,877 (12.0)	1.15 (1.08–1.21)	0	0	N/A	351 (12.2)	435 (15.8)	1.30 (1.14–1.49)

^a^Adjusted for age and sex

Abbreviations: ACEis, angiotensin-converting enzyme inhibitors; ARBs, angiotensin-receptor II blockers; CI, confidence interval; CKD: chronic kidney disease; eGFR, estimated glomerular filtration rate; HK;

hyperkalemia; IHD, ischemic heart disease; PR, prevalence ratio; RASi, renin angiotensin system inhibitors

### Potassium trajectories and treatment changes

**Fig 1:**
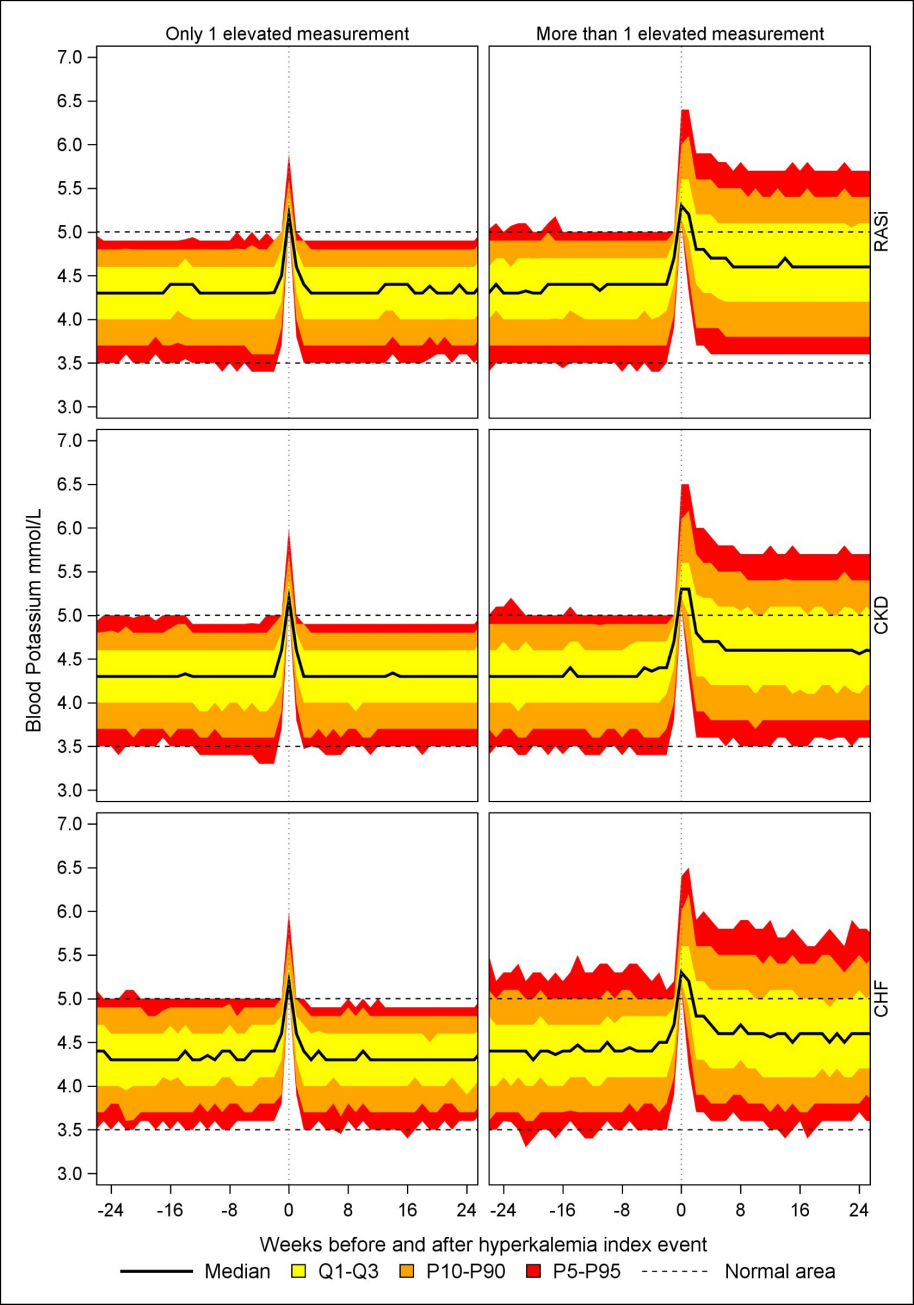
Median potassium levels (mmol/L) in the three patient cohorts before and after a first hyperkalemia event. **Data are shown separately for patients with one and more than one hyperkalemia event during the 6 months trajectory period.** Abbreviations: CHF, chronic heart failure; CKD, chronic kidney disease; RASi, renin angiotensin system inhibitors Patients were at risk of hyperkalemia before their first hyperkalemia event if they did not fulfil the criteria for chronic heart failure, chronic kidney disease, and RASi treatment, respectively.

When we evaluated median potassium levels in patients before and after a first hyperkalemia event (Fig 1), the median potassium level during the hyperkalemia event was similar in the three cohorts (around 5.3 mmol/L). In patients with a single hyperkalemia event, the median potassium levels returned to pre-hyperkalemia levels in approximately 2–4 weeks. In patients with repeated hyperkalemia, the reduction in potassium levels occurred more slowly and potassium did not reach the median pre-hyperkalemia level. As observed in the 75^th^-90^th^ percentiles and 90^th^-95^th^ percentiles, a considerable proportion of patients had median potassium levels above 5.0 mmol/L during the 6-month period.

**Fig 2:**
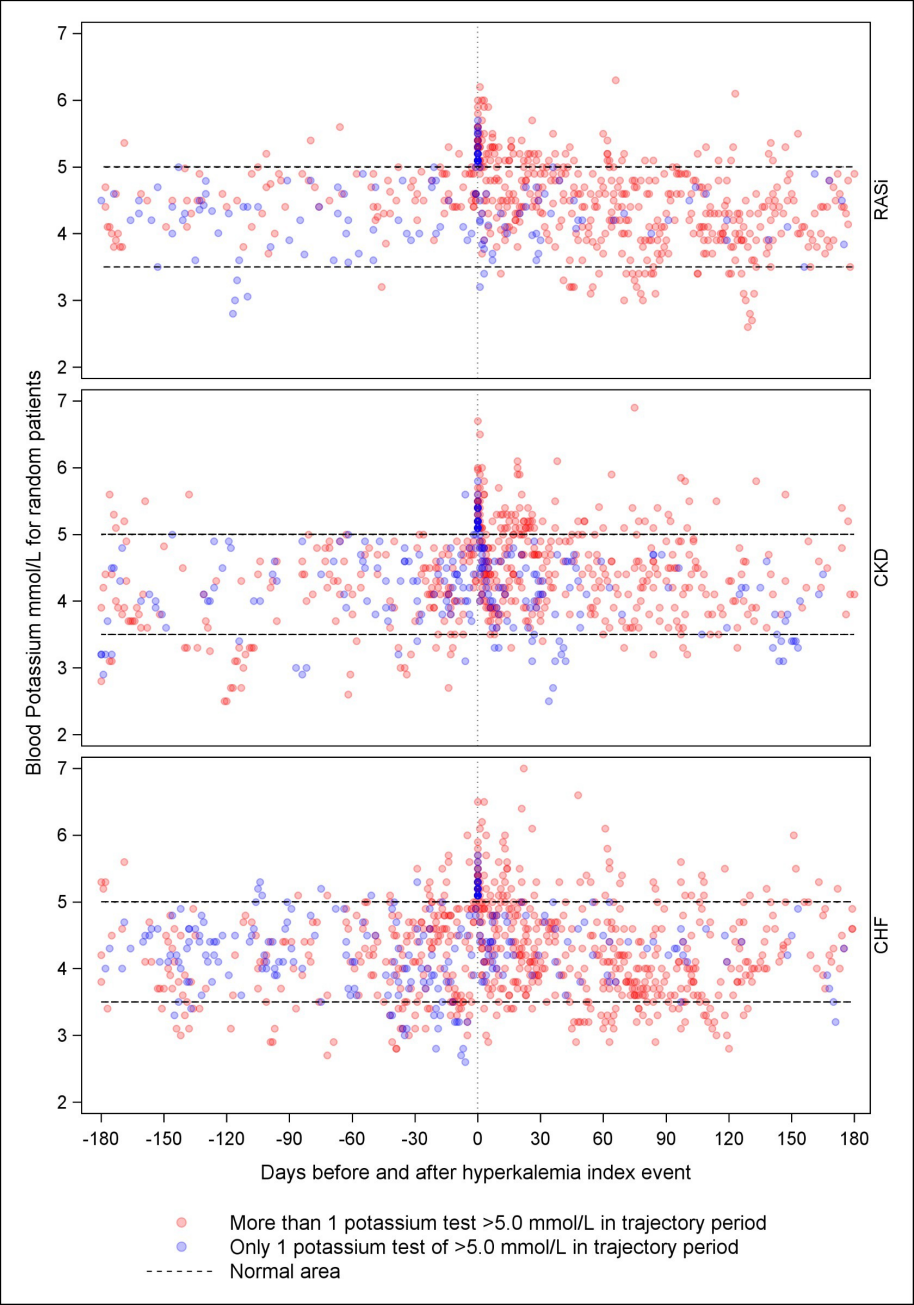
All potassium test results for 50 randomly sampled individuals in three cohorts before and after first hyperkalemia event, by patients with one and more than one hyperkalemia event. Abbreviations: CHF, chronic heart failure; CKD, chronic kidney disease; RASi, renin angiotensin system inhibitors Patients were at risk of hyperkalemia before their first hyperkalemia event if they did not fulfil the criteria for chronic heart failure, chronic kidney disease, and RASi treatment, respectively.

**Fig 3:**
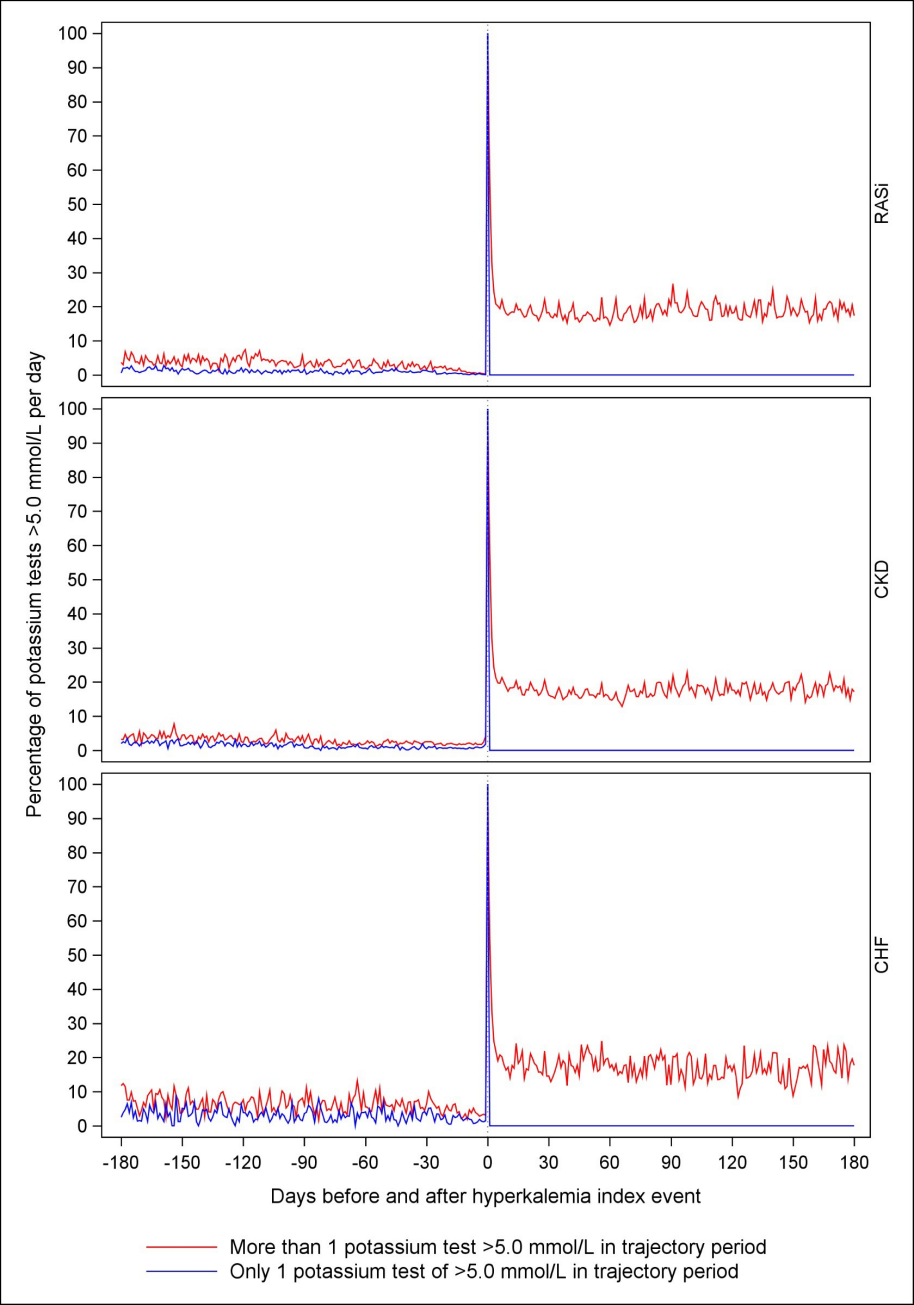
Proportion of potassium level tests results above 5.0 mmol/L in relation to total number of potassium test at the same day, before and after the first index hyperkalemia event. Abbreviations: CHF, chronic heart failure; CKD, chronic kidney disease; RASi, renin angiotensin system inhibitors Patients were at risk of hyperkalemia before their first hyperkalemia event if they did not fulfil the criteria for chronic heart failure, chronic kidney disease, and RASi treatment, respectively.

All potassium tests measured for 50 randomly selected patients within each of the cohorts indicated that patients in the repeated hyperkalemia group typically had several hyperkalemia events but also more hypokalemia events than patients without repeated hyperkalemia during the 6 month trajectory period (Fig 2), indicating greater potassium variability. The daily proportion of potassium test results >5.0 mmol/L reached 20% in the repeated hyperkalemia group and remained elevated throughout the entire 6 month trajectory period (Fig 3).

**Fig 4:**
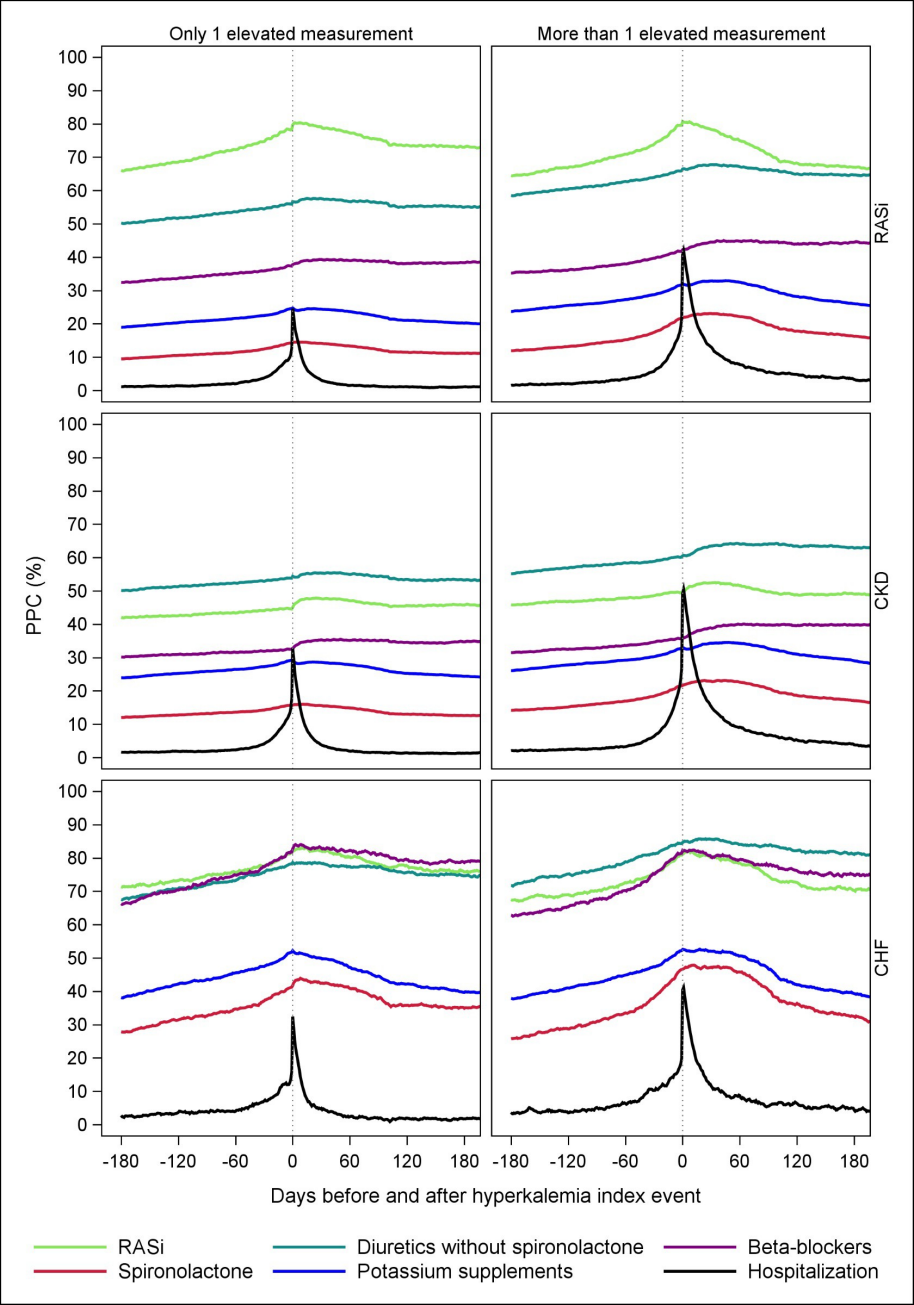
Proportion of patients hospitalized or covered with selected prescription drugs of interest before and after the first index hyperkalemia event, by patients with one and more than one hyperkalemia event. ^a^Although all patients enrolled in the RASi cohort received RASi treatment at cohort entry, only 80% of the patients received treatment at the time of hyperkalemia. ^b^Due to a low number of patients receiving dialysis before and after hyperkalemia (<1%), these data were omitted from the figure. Abbreviations: CHF, chronic heart failure; CKD, chronic kidney disease; PPC, proportion of patients covered; RASi, renin angiotensin system inhibitors.

The hospitalization rate ranged between 30%-50% at the time of the first hyperkalemia in the three cohorts (Fig 4), however this number decreased during follow-up. In all three cohorts there was an increase in the proportion of patients treated with RASi and spironolactone before the hyperkalemia event. A considerable proportion of the patients in both the RASi and CHF cohort discontinued RASi, spironolactone, and potassium supplements, while more patients required diuretics in the period following hyperkalemia. This pattern was not observed in the CKD cohort.

### Sensitivity analyses

Restricting the analyses to potassium measured at GP offices and to patients surviving, did not materially change the results of the predictors for repeated hyperkalemia (S5 and S6 Tables). The median levels of the highest weekly potassium measurements per individual and the proportions of potassium level test results >5.0 mmol/L during the 6 month trajectory period were higher when focusing solely on measurements performed at GP offices (S2–S5 Figs). Changing the definition from repeated to recurrent hyperkalemia and requiring at least two potassium tests during the trajectory period left the results broadly unchanged (S7 and S8 Tables).

## Discussion

Among RASi users, patients with CKD, and patients with CHF, predictors for repeated hyperkalemia included moderate to severe hyperkalemia, low eGFR, diabetes, and use of spironolactone. Following hyperkalemia, the decline in median potassium levels occurred throughout 2–4 weeks, after which median potassium levels stabilized at a normal level among patients with one hyperkalemia event. In patients with repeated hyperkalemia however, median potassium levels remained increased over time.

Although previous studies have suggested that hyperkalemia is frequent among patients with diabetes and heart failure,[1,4,5,13] our study adds to existing literature by elucidating predictors for repeated hyperkalemia and by reporting data on the evolution of potassium levels following a first hyperkalemia event in cohorts of clinical interest.

Consistent with previous findings, reduced kidney function and treatment with ACEis and spirolactone were predictive for development of hyperkalemia.[1,13] Importantly, we found that potassium ≥5.6 mmol/L as opposed to potassium <5.6 mmol/L predicted repeated hyperkalemia events, thus suggesting that a first mild hyperkalemia event is likely to represent a single event within 6 months, albeit even mild potassium elevations may be associated with cardiac arrhythmias and increased mortality.[14] Our findings indicate that potassium monitoring for users of RASi, CKD patients, and CHF patients requires more attention. Monitoring regimens may be intensified for patients presenting with moderate to severe hyperkalemia and also for patients with multiple predictors for repeated hyperkalemia. Awareness of and response to hyperkalemia may lead to a reduction in hyperkalemia-associated adverse outcomes, as indicated in a study of patients with diabetes recently initiating RASi therapy.[15] Potassium levels remained higher than before the hyperkalemia event over 2–4 weeks, and median levels were higher than the normal range for many patients with more than one hyperkalemia event. These findings indicate that more clinical attention on prevention strategies after hyperkalemia may be prudent in populations at high risk.

Interestingly, many patients had only one hyperkalemia event within 6 months, after which potassium levels returned to normal. This indicates that the underlying cause of hyperkalemia may have been eliminated, but it may also be attributed to the mild and transient elevated creatinine levels often observed after the start of RASi.[16] One explanation for the declining potassium levels in the RASi and CHF cohort over time was the reduction in patients receiving RASi and spironolactone, which may have reduced the potassium levels, but at the potential expense of exacerbating the underlying cardiovascular condition. The observed discontinuation in RASi and spironolactone use was delayed after the hyperkalemia event. This likely reflects that many patients had redeemed a prescription for these drugs soon before their hyperkalemia event, and discontinuation was not captured by the registry until patients had ceased to refill their prescription. In the CKD cohort, RASi therapy was relatively unchanged following hyperkalemia, suggesting that clinicians are less reluctant to prescribe RASi even to CKD patients with hyperkalemia. Future studies have the potential to support continuation of RASi treatments with effective chronic potassium management.

Among patients with a first hyperkalemia event, median potassium levels were higher in the primary care setting than in hospitalized patients. One explanation for this may be that patients followed for hyperkalemia in the primary care are those with slightly increased potassium levels, and thus are less likely to receive emergency treatment with rapid onset of action and corresponding rapid reduction in potassium levels. Although patients followed up by their GPs are less sick than hospitalized patients, our results underscore that clinical attention to hyperkalemia in these patients may be warranted to maintain physiological potassium levels.

One of the main strengths of this study lies in the population-based design made possible by the uniformly organized Danish healthcare system. This essentially eliminates selection bias, encountered in clinic- or health insurance-based epidemiological studies. Limitations of this study also merit consideration. Because only potassium measured during routine clinical practice was available *i*.*e*. when clinicians judged that a potassium test was indicated, we might have underestimated the true hyperkalemia burden. However, our study population consisted of patients with chronic comorbid conditions; that is, patients were all likely to receive regular blood tests in a uniform system with equal and free access to health care services, as also supported by a high number of potassium tests observed in our cohorts. Laboratory tests were non-standardized across the included hospitals. The frequency of pseudo-hyperkalemia was presumably rare, as laboratories usually take precautions to avoid false-positive potassium values, *e*.*g*. non-reporting of potassium values if hemolysis is observed. The size of the CHF cohort was considerably smaller than the CKD and RASi cohorts, as we applied a strict algorithm to ensure inclusion of true CHF patients. Although this may have lowered the sensitivity of the CHF diagnosis, prioritization of a high positive predictive value of study cohorts is generally recommended.[17]

## Conclusions

Among new-users of RASi and in patients with CKD or CHF, repeated hyperkalemia events were frequent, especially in patients with high elevated potassium, reduced kidney function, or in those with diabetes. After a first hyperkalemia event, median potassium levels reverted to normal after 2–4 weeks, but remained persistently high in patients with more than one hyperkalemia event. Clinical predictors can identify patients who may benefit from intensified monitoring and management of hyperkalemia.

## Supporting information

S1 Table Codes used to identify study variables. (DOCX)

S2 Table Baseline characteristics of the three patient cohorts; RASi new-users and in patients with chronic kidney or heart failure. (DOCX)

S3 Table Number of patients included in the three cohorts and the overlapping study populations. (DOCX)

S4 Table Prevalence of selected clinical predictors in patients with one and more than one hyperkalemia events during a 6-month trajectory period and corresponding prevalence ratios. (DOCX)

S5 Table Prevalence of clinical predictors in patients with one and more than one hyperkalemia events during a 6-month trajectory period and corresponding prevalence ratios, restricted to measurements at general practitioners. (DOCX)

S6 Table Prevalence of clinical predictors in patients with one and more than one hyperkalemia event during a 6-month trajectory period and corresponding prevalence ratios. Restricted to patients surviving in the 6-month trajectory period.
(DOCX)

S7 Table Prevalence of clinical predictors in patients with one and more than one hyperkalemia events during a 6-month trajectory period and corresponding prevalence ratios, changing the definition from repeated to recurrent hyperkalemia. (DOCX)

S8 Table Prevalence of clinical predictors in patients with one and more than one hyperkalemia events during a 6-month trajectory period and corresponding prevalence ratios, requiring at least two potassium tests during the trajectory period. (DOCX)

S1 Fig Time frame of the cohort study with examples of the different cohort entries. A. The overall study design, illustrating the 6 month trajectory period following hyperkalemia. B. One patient enrolled in the RASi new user cohort and subsequently in the CKD cohort. C. One patient enrolled in the CHF cohort at the time of fulfilling all the CHF criteria: echocardiography, hospitalization with CHF, redeemed prescriptions of ACEi and beta blocker.
(DOCX)

S2 Fig Median potassium levels (mmol/L) in the three patient cohorts before and after a first hyperkalemia event, restricted to measurements at general practitioners. (DOCX)

S3 Fig All potassium test results for 50 randomly sampled individuals in three cohorts before and after first hyperkalemia event, by patients with one and more than one hyperkalemia event, according to measurements at general practitioners. (DOCX)

S4 Fig Proportion of potassium level tests results above 5.0 mmol/L in relation to total number of potassium test at the same day, before and after the first index hyperkalemia event, according to measurements at general practitioners. (DOCX)

S5 Fig Proportion of patients hospitalized or covered with selected prescription drugs of interest before and after the first index hyperkalemia event, by patients with one and more than one hyperkalemia event, according to measurements at general practitioners. (DOCX)
