# Supplementary material for: Predictors for repeated hyperkalemia and potassium trajectories in high-risk patients — A population-based cohort study
Source: PLoS One. 2019 Jun 21;14(6):e0218739. doi: 10.1371/journal.pone.0218739 (PMC6588240; doi:10.1371/journal.pone.0218739)
Supplement: S1 Table — (DOCX) [file pone.0218739.s001.docx]

| **S1 Table. Codes used to identify study variables.** | |
| --- | --- |
| **Study populations** | **Algorithm** |
| RASi | ATC: C09A-D |
| Chronic kidney disease | Defined as either a previous hospitalization with a hospital diagnosis of chronic nephritic syndrome (N03), glomerular disease (N08), chronic kidney disease / chronic renal failure (N18-N19), diabetic nephropathy (E102, E112, E122, E132, E142), or hypertension with renal failure (I120, I131, I132); or a previous dialysis procedure (BJFD2); or presence of two occurrences more than 90 days apart of a creatinine measurement corresponding to an eGFR <60 mL/min/1.73m2 |
| Heart failure | ICD-10: I50, I110, I130, I132 |
| **Hyperkalemia** | Code NPU03230. Analysis numbers AAA00958, 110262, 111262 115230, 115231, 1511140, 1610147, 1613230, 1710304, 1713230, 1813230, 1817159, 1311140, 1411140 |
| **Covariates** |  |
| Diabetes | Defined as a previous hospitalization with a hospital diagnosis of diabetes (ICD-8 259-250; ICD-10 E10-E14, H360), or at least one previous prescription for a glucose-lowering drug (ATC code A10), or presence of two occurrences of a HbA1c measurement > 6.5% |
| Ischemic heart disease | ICD-10: I20-I25 |
| Hypertension | Defined as a previous hospitalization with a hospital diagnosis of hypertension (I10-I15) or a previous prescription for at least two of the following classes of antihypertensive drugs: adrenergic blockers and others (C02), non-loop diuretics (C03A, C03B, C03D, C03E), β blockers (C07), calcium channel blockers (C08), RAS inhibitors (C09) |
| Atrial fibrillation or flutter | ICD-10: I48 |
| Valvular heart disease | ICD-10: I00-I02, I05-I09, I34, I35, I36, I37, Q20-Q25, Q22, Q23 |
| Cardiomyopathy | ICD-10: I42 |
| Peripheral vascular disease | ICD-10: I70; I71; I72; I73; I74; I77 |
| Cerebrovascular disease | ICD-10: I60-I69; G45; G46 |
| Dementia | ICD-10: F00-F03, F05.1, G30 |
| Chronic pulmonary disease | ICD-10: J40-J47; J60-J67; J68.4; J70.1; J70.3; J84.1; J92.0; J96.1; J98.2; J98.3 |
| Connective tissue disease | ICD-10: M05, M06, M08, M09, M30, M31, M32, M33, M34, M35, M36, D86 |
| Peptic ulcer disease | ICD-10: K22.1; K25-K28 |
| Any cancer | ICD-10: C00-C96 |
| Alcoholism-related diseases | ICD-10: T36-T65; F10-F19; G312; G621; G721; I 426; K292; K860; K70; R780; T51; Z714; Z721 |
| Obesity | ICD-10: E65-E66 |
| **Medication** |  |
| ACEis | ATC: C09A, C09B |
| ARBs | ATC: C09C, C09D |
| Spironolactone | ATC: C03D, C03E |
| Macrolides | ATC: J01FA |
| Beta blockers | ATC: C07 |
| Azoles | ATC: J02A |
| Digoxin | ATC: C01AA05 |
| Non-steroidal anti-inflammatory drugs | ATC:M01A |
| Potassium supplements | ATC: A12B |
| Trimethoprim | ATC: J01EA, J01EE |
| Loop diuretics | ATC: C03C |
| **Other codes** |  |
| Echocardiography | UXUC80 |

Abbreviations: ACEis, angiotensin-converting enzyme inhibitors; ARBs, angiotensin-receptor II blockers; ATC, anatomical therapeutic chemical classification; ICD, international classification of diseases; RASi, renin angiotensin system inhibitors
