# Supplementary material for: Predictors for repeated hyperkalemia and potassium trajectories in high-risk patients — A population-based cohort study
Source: PLoS One. 2019 Jun 21;14(6):e0218739. doi: 10.1371/journal.pone.0218739 (PMC6588240; doi:10.1371/journal.pone.0218739)
Supplement: S2 Table — (DOCX) [file pone.0218739.s002.docx]

| **S2 Table**. **Baseline characteristics of the three patient cohorts; RASi new-users and in patients with chronic kidney or heart failure.** | | | |
| --- | --- | --- | --- |
|  | **RASi new-users** | **Chronic kidney disease** | **Chronic heart failure** |
| **Total** | 262,375 | 157,283 | 14,600 |
| **Women** | 130,373 (49.7) | 92,558 (58.8) | 5,360 (36.7) |
| **Median age (25^th^–75^th^ percentile)** | 62.9 (53.3-72.7) | 72.6 (63.2-80.6) | 72.8 (63.5-80.2) |
| **Year of diagnosis** |  |  |  |
| 2000–2006 | 135,839 (51.8) | 87,223 (55.5) | 5,631 (38.6) |
| 2007–2012 | 126,536 (48.2) | 70,060 (44.5) | 8,969 (61.4) |
| **eGFR groups (mL/min/1.73m^2^)** |  |  |  |
| Not measured | 52,058 (19.8) | 2,452 (1.6) | 2,918 (20.0) |
| ≥60 | 139,871 (53.3) | 1,600 (1.0) | 3,256 (22.3) |
| 45–59 | 47,299 (18.0) | 108,715 (69.1) | 3,117 (21.3) |
| 30–44 | 15,763 (6.0) | 31,322 (19.9) | 2,690 (18.4) |
| 15–29 | 5,377 (2.0) | 9,724 (6.2) | 1,852 (12.7) |
| <15 | 1,453 (0.6) | 3,060 (1.9) | 564 (3.9) |
| Dialysis | 554 (0.2) | 410 (0.3) | 203 (1.4) |
| **Comorbidities** |  |  |  |
| Diabetes | 37,080 (14.1) | 23,149 (14.7) | 3,438 (23.5) |
| Chronic kidney disease | 44,333 (16.9) | N/A | 6,333 (43.4) |
| Heart failure | 13,320 (5.1) | 13,823 (8.8) | N/A |
| Ischemic heart disease | 28,000 (10.7) | 23,173 (14.7) | 8,288 (56.8) |
| Hypertension | 49,009 (18.7) | 35,171 (22.4) | 6,558 (44.9) |
| Atrial fibrillation or flutter | 16,277 (6.2) | 17,812 (11.3) | 5,954 (40.8) |
| Valvular heart disease | 7,692 (2.9) | 7,351 (4.7) | 2,622 (18.0) |
| Cardiomyopathy | 2,562 (1.0) | 2,052 (1.3) | 2,012 (13.8) |
| Peripheral vascular disease | 10,399 (4.0) | 10,681 (6.8) | 1,802 (12.3) |
| Cerebrovascular disease | 22,767 (8.7) | 19,576 (12.4) | 2,224 (15.2) |
| Dementia | 1,142 (0.4) | 2,198 (1.4) | 137 (0.9) |
| Chronic pulmonary disease | 17,637 (6.7) | 16,123 (10.3) | 2,432 (16.7) |
| Connective tissue disease | 7,368 (2.8) | 6,608 (4.2) | 683 (4.7) |
| Peptic ulcer disease | 10,293 (3.9) | 9,740 (6.2) | 1,123 (7.7) |
| Any cancer | 19,483 (7.4) | 23,567 (15.0) | 1,691 (11.6) |
| Alcoholism-related disorders | 13,737 (5.2) | 9,563 (6.1) | 1,276 (8.7) |
| Obesity | 12,225 (4.7) | 7,044 (4.5) | 1,309 (9.0) |
| **Medication within past 6 months** |  |  |  |
| ACEis | 192,065 (73.2) | 33,284 (21.2) | 10,871 (74.5) |
| ARBs | 70,571 (26.9) | 20,805 (13.2) | 3,779 (25.9) |
| Spironolactone | 10,630 (4.1) | 11,405 (7.3) | 4,289 (29.4) |
| Macrolides | 11,101 (4.2) | 0 (0.0) | 1,197 (8.2) |
| Beta blockers | 63,346 (24.1) | 37,146 (23.6) | 12,451 (85.3) |
| Azoles | 3,200 (1.2) | 2,228 (1.4) | 219 (1.5) |
| Digoxin | 8,227 (3.1) | 7,379 (4.7) | 3,110 (21.3) |
| NSAIDs | 49,411 (18.8) | 26,728 (17.0) | 2,148 (14.7) |
| Potassium supplements | 23,869 (9.1) | 23,900 (15.2) | 6,609 (45.3) |
| Trimethoprim | 1,665 (0.6) | 2,673 (1.7) | 218 (1.5) |
| Loop diuretics | 28,327 (10.8) | 31,905 (20.3) | 10,452 (71.6) |
| Values are counts (%), unless stated otherwise.  Abbreviations: ACEis, angiotensin-converting enzyme inhibitors; ARBs, angiotensin-receptor II blockers; eGFR, estimated glomerular filtration rate; NSAIDs, non-steroidal anti-inflammatory drugs; RASi, renin angiotensin system inhibitors. | | | |
