# Supplementary material for: Predictors for repeated hyperkalemia and potassium trajectories in high-risk patients — A population-based cohort study
Source: PLoS One. 2019 Jun 21;14(6):e0218739. doi: 10.1371/journal.pone.0218739 (PMC6588240; doi:10.1371/journal.pone.0218739)
Supplement: S3 Table — (DOCX) [file pone.0218739.s003.docx]

| **S3 Table**. **Number of patients included in the three cohorts and the overlapping study populations.** | | | |
| --- | --- | --- | --- |
| **RASi new-users** | **Chronic kidney disease** | **Chronic heart failure** | **No. (%)** |
| Included in the cohort |  |  | 179,966 (52.4) |
|  | Included in the cohort |  | 79,235 (23.1) |
|  |  | Included in the cohort | 759 (0.22) |
| Included in the cohort | Included in the cohort |  | 69,686 (20.3) |
| Included in the cohort |  | Included in the cohort | 5,479 (1.6) |
|  | Included in the cohort | Included in the cohort | 1,118 (0.3) |
| Included in the cohort | Included in the cohort | Included in the cohort | 7,244 (2.1) |
| Abbreviations: RASi, renin angiotensin system inhibitors | | | |
