# Supplementary material for: Predictors for repeated hyperkalemia and potassium trajectories in high-risk patients — A population-based cohort study
Source: PLoS One. 2019 Jun 21;14(6):e0218739. doi: 10.1371/journal.pone.0218739 (PMC6588240; doi:10.1371/journal.pone.0218739)
Supplement: S4 Table — (DOCX) [file pone.0218739.s004.docx]

| **S4 Table**. **Prevalence of selected clinical predictors in patients with one and more than one hyperkalemia events during a 6-month trajectory period and corresponding prevalence ratios.** | | | | | | | | | |
| --- | --- | --- | --- | --- | --- | --- | --- | --- | --- |
|  | **RASi new-users** | | | **Chronic kidney disease** | | | **Chronic heart failure** | | |
|  | **1 HK event, n (%)** | **≥2 HK events, n (%)** | **PR^a^**  **(95% CI)** | **1 HK event, n (%)** | **≥2 HK events, n (%)** | **PR^a^**  **(95% CI)** | **1 HK event, n (%)** | **≥2 HK events, n (%)** | **PR^a^**  **(95% CI)** |
| **Total** | 26,164 (100) | 15,654 (100) | N/A | 26,257 (100) | 17,588 (100) | N/A | 2,885 (100) | 2,749 (100) | N/A |
| **Log (eGFR)** |  |  |  |  |  |  |  |  |  |
| Above Q3 | 7,708 (29.5) | 2,500 (16.0) | 0.57 (0.54-0.59) | 7,693 (29.3) | 3,435 (19.5) | 0.65 (0.63-0.67) | 813 (28.2) | 473 (17.2) | 0.62 (0.56-0.68) |
| Median-Q3 | 6,925 (26.5) | 3,273 (20.9) | 0.79 (0.76-0.82) | 6,845 (26.1) | 3,743 (21.3) | 0.82 (0.79-0.85) | 704 (24.4) | 634 (23.1) | 0.94 (0.86-1.04) |
| Q1-Median | 6,182 (23.6) | 4,268 (27.3) | 1.11 (1.07-1.15) | 6,316 (24.1) | 4,580 (26.0) | 1.10 (1.07-1.14) | 614 (21.3) | 691 (25.1) | 1.16 (1.06-1.28) |
| Below Q1 | 4,854 (18.6) | 5,333 (34.1) | 1.79 (1.74-1.86) | 5,324 (20.3) | 5,664 (32.2) | 1.59 (1.54-1.64) | 511 (17.7) | 785 (28.6) | 1.60 (1.45-1.76) |
| **Comorbidities** |  |  |  |  |  |  |  |  |  |
| Hypertension | 22,946 (87.7) | 14,309 (91.4) | 1.04 (1.03-1.04) | 17,945 (68.3) | 12,933 (73.5) | 1.08 (1.07-1.09) | 2,878 (99.8) | 2,745 (99.9) | 1.00 (1.00-1.00) |
| Atrial fibrillation or flutter | 4,459 (17.0) | 3,466 (22.1) | 1.23 (1.18-1.27) | 4,966 (18.9) | 3,793 (21.6) | 1.15 (1.11-1.19) | 1,317 (45.6) | 1,298 (47.2) | 1.02 (0.97-1.08) |
| Valvular heart disease | 2,161 (8.3) | 1,710 (10.9) | 1.28 (1.21-1.36) | 2,127 (8.1) | 1,763 (10.0) | 1.25 (1.18-1.33) | 619 (21.5) | 636 (23.1) | 1.07 (0.97-1.18) |
| Cardiomyopathy | 811 (3.1) | 600 (3.8) | 1.27 (1.15-1.41) | 686 (2.6) | 560 (3.2) | 1.12 (1.01-1.26) | 482 (16.7) | 418 (15.2) | 0.92 (0.82-1.04) |
| Peripheral vascular disease | 2,893 (11.1) | 2,215 (14.1) | 1.24 (1.18-1.31) | 3,049 (11.6) | 2,408 (13.7) | 1.16 (1.11-1.22) | 507 (17.6) | 570 (20.7) | 1.18 (1.06-1.31) |
| Cerebrovascular disease | 4,595 (17.6) | 2,954 (18.9) | 1.03 (0.99-1.08) | 4,867 (18.5) | 3,137 (17.8) | 0.96 (0.92-1.00) | 542 (18.8) | 551 (20.0) | 1.06 (0.95-1.18) |
| Dementia | 461 (1.8) | 231 (1.5) | 0.77 (0.66-0.91) | 666 (2.5) | 299 (1.7) | 0.71 (0.62-0.82) | 32 (1.1) | 36 (1.3) | 1.15 (0.72-1.85) |
| Chronic pulmonary disease | 3,887 (14.9) | 2,856 (18.2) | 1.21 (1.16-1.26) | 4,393 (16.7) | 3,433 (19.5) | 1.16 (1.12-1.21) | 633 (21.9) | 675 (24.6) | 1.12 (1.02-1.23) |
| Connective tissue disease | 1,299 (5.0) | 896 (5.7) | 1.16 (1.06-1.26) | 1,486 (5.7) | 996 (5.7) | 1.04 (0.96-1.13) | 197 (6.8) | 168 (6.1) | 0.89 (0.73-1.09) |
| Peptic ulcer disease | 2,180 (8.3) | 1,598 (10.2) | 1.20 (1.12-1.27) | 2,645 (10.1) | 1,824 (10.4) | 1.03 (0.97-1.09) | 312 (10.8) | 329 (12.0) | 1.10 (0.95-1.27) |
| Any cancer | 4,149 (15.9) | 3,200 (20.4) | 1.25 (1.20-1.31) | 5,378 (20.5) | 4,321 (24.6) | 1.20 (1.16-1.24) | 414 (14.4) | 465 (16.9) | 1.17 (1.03-1.32) |
| Alcoholism-related disorders | 2,309 (8.8) | 1,501 (9.6) | 1.13 (1.07-1.21) | 2,291 (8.7) | 1,868 (10.6) | 1.13 (1.07-1.20) | 308 (10.7) | 284 (10.3) | 0.98 (0.84-1.14) |
| Obesity | 1,932 (7.4) | 1,393 (8.9) | 1.27 (1.19-1.35) | 1,624 (6.2) | 1,362 (7.7) | 1.23 (1.15-1.32) | 294 (10.2) | 296 (10.8) | 1.07 (0.92-1.24) |
| **Comedication** |  |  |  |  |  |  |  |  |  |
| Beta blockers | 11,482 (43.9) | 7,605 (48.6) | 1.10 (1.07-1.12) | 9,321 (35.5) | 6,837 (38.9) | 1.09 (1.07-1.12) | 2,508 (86.9) | 2,440 (88.8) | 1.02 (1.00-1.04) |
| Azoles | 796 (3.0) | 598 (3.8) | 1.30 (1.17-1.44) | 929 (3.5) | 705 (4.0) | 1.17 (1.06-1.28) | 87 (3.0) | 99 (3.6) | 1.19 (0.90-1.58) |
| Digoxin | 2,306 (8.8) | 1,868 (11.9) | 1.27 (1.20-1.35) | 2,487 (9.5) | 1,976 (11.2) | 1.22 (1.16-1.29) | 791 (27.4) | 739 (26.9) | 0.97 (0.89-1.06) |
| NSAIDs | 7,648 (29.2) | 4,740 (30.3) | 1.04 (1.01-1.08) | 7,090 (27.0) | 4,925 (28.0) | 1.05 (1.01-1.08) | 600 (20.8) | 630 (22.9) | 1.10 (1.00-1.22) |
| Potassium supplements | 7,113 (27.2) | 5,434 (34.7) | 1.23 (1.19-1.26) | 7,675 (29.2) | 5,830 (33.1) | 1.17 (1.14-1.21) | 1,599 (55.4) | 1,563 (56.9) | 1.02 (0.97-1.07) |
| Trimethoprim | 841 (3.2) | 652 (4.2) | 1.24 (1.12-1.37) | 1,103 (4.2) | 769 (4.4) | 1.10 (1.01-1.21) | 85 (2.9) | 95 (3.5) | 1.15 (0.86-1.53) |
| Loop diuretics | 8,651 (33.1) | 7,399 (47.3) | 1.38 (1.35-1.42) | 9,155 (34.9) | 7,622 (43.3) | 1.27 (1.24-1.30) | 2,322 (80.5) | 2,356 (85.7) | 1.06 (1.04-1.09) |
| ^a^Adjusted for age and sex  Abbreviations: ACEis, angiotensin-converting enzyme inhibitors; ARBs, angiotensin-receptor II blockers; CI, confidence interval; CKD: chronic kidney disease; eGFR, estimated Glomerular Filtration Rate; HK, hyperkalemia; NSAIDs, non-steroidal anti-inflammatory drugs; PR, prevalence ratio; RASi, renin angiotensin system inhibitors | | | | | | | | | |
