# Supplementary material for: Predictors for repeated hyperkalemia and potassium trajectories in high-risk patients — A population-based cohort study
Source: PLoS One. 2019 Jun 21;14(6):e0218739. doi: 10.1371/journal.pone.0218739 (PMC6588240; doi:10.1371/journal.pone.0218739)
Supplement: S5 Table — (DOCX) [file pone.0218739.s005.docx]

| **S5 Table**. **Prevalence of clinical predictors in patients with one and more than one hyperkalemia events during a 6-month trajectory period and corresponding prevalence ratios, restricted to measurements at general practitioners.** | | | | | | | | | |
| --- | --- | --- | --- | --- | --- | --- | --- | --- | --- |
|  | **RASi new-users** | | | **Chronic kidney disease** | | | **Chronic heart failure** | | |
|  | **1 HK event, n (%)** | **≥2 HK events, n (%)** | **PR^a^**  **(95% CI)** | **1 HK event, n (%)** | **≥2 HK events, n (%)** | **PR^a^**  **(95% CI)** | **1 HK event, n (%)** | **≥2 HK events, n (%)** | **PR^a^**  **(95% CI)** |
| **Total** | 23,036 (100) | 9,047 (100) |  | 21,629 (100) | 8,837 (100%) |  | 2,533 (100) | 1,578 (100%) |  |
| **Median (range) potassium tests 6 months before** | 2.0 (0.0-4.0) | 3.0 (1.0-8.0) |  | 2.0 (0.0-5.0) | 3.0 (1.0-9.0) |  | 5.0 (1.0-10.0) | 7.0 (2.0-14.0) |  |
| **Median (range) potassium tests 6 months after** | 3.0 (2.0-5.0) | 6.0 (4.0-13.0) |  | 3.00 (2.0-6.0) | 7.0 (4.0-14.0) |  | 4.0 (2.0-9.0) | 8.0 (5.0-15.0) |  |
| **Females** | 10,724 (46.6) | 3,979 (44.0) | 0.94 (0.91-0.96) | 11,620 (53.7) | 4,027 (45.6) | 0.86 (0.84-0.88) | 937 (37.0) | 572 (36.2) | 0.99 (0.91-1.07) |
| **Median age (range)** | 72.4 (62.6-80.7) | 73.8 (64.2-81.0) |  | 76.09 (67.04-83.15) | 74.8 (65.8-81.7) |  | 75.2 (67.2-81.9) | 75.1 (67.4-81.4) |  |
| **First-time K+ level (mmol/L)** |  |  |  |  |  |  |  |  |  |
| >5.0–5.5 | 20,531 (89.1) | 7,192 (79.5) | 0.89 (0.88-0.90) | 18,928 (87.5) | 6,906 (78.1) | 0.89 (0.88-0.90) | 2,192 (86.5) | 1,226 (77.7) | 0.90 (0.87-0.93) |
| 5.6–6.0 | 1,963 (8.5) | 1,421 (15.7) | 1.84 (1.73-1.96) | 2,079 (9.6) | 1,486 (16.8) | 1.75 (1.65-1.86) | 273 (10.8) | 275 (17.4) | 1.62 (1.39-1.89) |
| 6.1–6.5 | 331 (1.4) | 329 (3.6) | 2.52 (2.16-2.93) | 386 (1.8) | 337 (3.8) | 2.16 (1.87-2.50) | 45 (1.8) | 61 (3.9) | 2.18 (1.49-3.18) |
| 6.6–7.0 | 120 (0.5) | 73 (0.8) | 1.53 (1.14-2.05) | 135 (0.6) | 75 (0.8) | 1.37 (1.03-1.81) | 14 (0.6) | 9 (0.6) | 1.04 (0.45-2.38) |
| >7.0 | 91 (0.4) | 32 (0.4) | 0.90 (0.60-1.35) | 101 (0.5) | 33 (0.4) | 0.84 (0.56-1.24) | 9 (0.4) | 7 (0.4) | 1.26 (0.47-3.39) |
| **eGFR groups (mL/min/1.73m2)** |  |  |  |  |  |  |  |  |  |
| Not measured | 420 (1.8) | 172 (1.9) | 1.05 (0.88-1.25) | 36 (0.2) | 34 (0.4) | 2.14 (1.34-3.41) | 230 (9.1) | 159 (10.1) | 1.11 (0.91-1.34) |
| ≥60 | 7,054 (30.6) | 1,536 (17.0) | 0.55 (0.52-0.58) | 119 (0.6) | 65 (0.7) | 0.88 (0.64-1.22) | 313 (12.4) | 119 (7.5) | 0.58 (0.48-0.70) |
| 45–59 | 6,152 (26.7) | 1,893 (20.9) | 0.78 (0.75-0.82) | 9,347 (43.2) | 2,613 (29.6) | 0.67 (0.65-0.70) | 513 (20.3) | 265 (16.8) | 0.82 (0.72-0.94) |
| 30–44 | 5,251 (22.8) | 2,445 (27.0) | 1.17 (1.12-1.22) | 7,020 (32.5) | 2,937 (33.2) | 1.05 (1.02-1.09) | 707 (27.9) | 418 (26.5) | 0.96 (0.86-1.06) |
| 15–29 | 3,149 (13.7) | 1,904 (21.0) | 1.52 (1.44-1.60) | 3,870 (17.9) | 2,055 (23.3) | 1.33 (1.27-1.39) | 553 (21.8) | 396 (25.1) | 1.16 (1.04-1.30) |
| <15 | 807 (3.5) | 677 (7.5) | 2.14 (1.94-2.37) | 1,003 (4.6) | 766 (8.7) | 1.80 (1.64-1.97) | 168 (6.6) | 129 (8.2) | 1.24 (1.00-1.55) |
| Dialysis | 203 (0.9) | 420 (4.6) | 5.18 (4.39-6.10) | 234 (1.1) | 367 (4.2) | 3.27 (2.77-3.85) | 49 (1.9) | 92 (5.8) | 2.90 (2.07-4.08) |
| **Comorbidities** |  |  |  |  |  |  |  |  |  |
| Diabetes | 7,098 (30.8) | 3,222 (35.6) | 1.16 (1.12-1.20) | 5,392 (24.9) | 2,806 (31.8) | 1.23 (1.19-1.28) | 788 (31.1) | 578 (36.6) | 1.17 (1.08-1.28) |
| Chronic kidney disease | 12,221 (53.1) | 5,943 (65.7) | 1.23 (1.20-1.25) | 18,718 (86.5) | 7,625 (86.3) | 1.00 (0.99-1.01) | 1,696 (67.0) | 1,195 (75.7) | 1.14 (1.10-1.18) |
| Heart failure | 3,953 (17.2) | 2,069 (22.9) | 1.30 (1.24-1.36) | 3,836 (17.7) | 1,923 (21.8) | 1.23 (1.18-1.30) | 2,531 (99.9) | 1,575 (99.8) | 1.00 (1.00-1.00) |
| Ischemic heart disease | 6,074 (26.4) | 2,585 (28.6) | 1.05 (1.01-1.10) | 5,257 (24.3) | 2,384 (27.0) | 1.09 (1.04-1.13) | 1,639 (64.7) | 984 (62.4) | 0.96 (0.92-1.01) |
| Hypertension | 20,163 (87.5) | 8,206 (90.7) | 1.03 (1.03-1.04) | 15,310 (70.8) | 6,923 (78.3) | 1.11 (1.10-1.13) | 2,533 (100) | 1,576 (99.9) | 1.00 (1.00-1.00) |
| Atrial fibrillation or flutter | 3,784 (16.4) | 1,850 (20.4) | 1.21 (1.15-1.27) | 3,973 (18.4) | 1,790 (20.3) | 1.13 (1.07-1.19) | 1,162 (45.9) | 709 (44.9) | 0.98 (0.92-1.05) |
| Valvular heart disease | 1,900 (8.2) | 981 (10.8) | 1.30 (1.21-1.40) | 1,775 (8.2) | 900 (10.2) | 1.26 (1.17-1.36) | 544 (21.5) | 360 (22.8) | 1.07 (0.95-1.20) |
| Cardiomyopathy | 681 (3.0) | 379 (4.2) | 1.41 (1.25-1.60) | 576 (2.7) | 315 (3.6%) | 1.21 (1.06-1.39) | 421 (16.6) | 253 (16.0) | 0.94 (0.81-1.08) |
| Peripheral vascular disease | 2,502 (10.9) | 1,185 (13.1) | 1.19 (1.11-1.26) | 2,477 (11.5) | 1,176 (13.3) | 1.15 (1.08-1.23) | 439 (17.3) | 300 (19.0) | 1.10 (0.96-1.25) |
| Cerebrovascular disease | 3,891 (16.9) | 1,587 (17.5) | 1.02 (0.97-1.07) | 3,833 (17.7) | 1,528 (17.3) | 0.98 (0.93-1.04) | 445 (17.6) | 283 (17.9) | 1.03 (0.90-1.17) |
| Dementia | 362 (1.6) | 108 (1.2) | 0.74 (0.60-0.92) | 498 (2.3) | 107 (1.2) | 0.58 (0.47-0.72) | 22 (0.9) | 15 (1.0) | 1.12 (0.58-2.15) |
| Chronic pulmonary disease | 3,281 (14.2) | 1,401 (15.5) | 1.08 (1.02-1.14) | 3,392 (15.7) | 1,441 (16.3) | 1.04 (0.98-1.10) | 561 (22.1) | 326 (20.7) | 0.93 (0.83-1.05) |
| Connective tissue disease | 1,135 (4.9) | 484 (5.3) | 1.10 (0.99-1.22) | 1,243 (5.7) | 506 (5.7) | 1.04 (0.94-1.14) | 152 (6.0) | 79 (5.0) | 0.84 (0.64-1.09) |
| Peptic ulcer disease | 1,926 (8.4) | 894 (9.9) | 1.17 (1.08-1.26) | 2,139 (9.9) | 915 (10.4) | 1.06 (0.98-1.14) | 275 (10.9) | 167 (10.6) | 0.98 (0.82-1.18) |
| Any cancer | 3,487 (15.1) | 1,656 (18.3) | 1.19 (1.13-1.26) | 4,154 (19.2) | 1,982 (22.4) | 1.17 (1.12-1.23) | 336 (13.3) | 245 (15.5) | 1.18 (1.01-1.37) |
| Alcoholism-related disorders | 1,973 (8.6) | 772 (8.5) | 1.00 (0.92-1.08) | 1,820 (8.4) | 785 (8.9) | 0.96 (0.89-1.04) | 262 (10.3) | 159 (10.1) | 0.95 (0.79-1.14) |
| Obesity | 1,745 (7.6) | 780 (8.6) | 1.16 (1.07-1.26) | 1,392 (6.4) | 698 (7.9) | 1.19 (1.09-1.30) | 249 (9.8) | 176 (11.2) | 1.11 (0.92-1.33) |
| **Comedication** |  |  |  |  |  |  |  |  |  |
| ACEis | 15,540 (67.5) | 6,359 (70.3) | 1.04 (1.02-1.06) | 8,200 (37.9) | 4,029 (45.6) | 1.19 (1.15-1.22) | 1,834 (72.4) | 1,175 (74.5) | 1.03 (0.99-1.07) |
| ARBs | 7,081 (30.7) | 2,795 (30.9) | 1.01 (0.98-1.05) | 4,073 (18.8) | 1,931 (21.9) | 1.16 (1.11-1.22) | 578 (22.8) | 365 (23.1) | 1.01 (0.90-1.13) |
| Spironolactone | 4,196 (18.2) | 2,303 (25.5) | 1.39 (1.33-1.45) | 3,996 (18.5) | 2,096 (23.7) | 1.31 (1.25-1.37) | 1,266 (50.0) | 826 (52.3) | 1.05 (0.98-1.11) |
| Macrolides | 2,348 (10.2) | 1,008 (11.1) | 1.10 (1.03-1.18) | 0 | 0 |  | 323 (12.8) | 214 (13.6) | 1.06 (0.91-1.25) |
| Beta blockers | 10,348 (44.9) | 4,615 (51.0) | 1.13 (1.10-1.16) | 8,189 (37.9) | 3,826 (43.3) | 1.14 (1.11-1.17) | 2,181 (86.1) | 1,350 (85.6) | 0.99 (0.97-1.02) |
| Azoles | 662 (2.9) | 280 (3.1) | 1.10 (0.96-1.26) | 743 (3.4) | 291 (3.3) | 0.98 (0.85-1.12) | 81 (3.2) | 44 (2.8) | 0.87 (0.61-1.25) |
| Digoxin | 2,036 (8.8) | 1,042 (11.5) | 1.26 (1.18-1.36) | 2,100 (9.7) | 945 (10.7) | 1.17 (1.09-1.25) | 703 (27.8) | 394 (25.0) | 0.90 (0.81-1.01) |
| NSAIDs | 6,724 (29.2) | 2,643 (29.2) | 1.01 (0.97-1.05) | 5,903 (27.3) | 2,444 (27.7) | 1.02 (0.98-1.06) | 521 (20.6) | 355 (22.5) | 1.10 (0.97-1.23) |
| Potassium supplements | 6,120 (26.6) | 2,741 (30.3) | 1.12 (1.08-1.17) | 6,222 (28.8) | 2,606 (29.5) | 1.08 (1.04-1.12) | 1,399 (55.2) | 798 (50.6) | 0.92 (0.87-0.98) |
| Trimethoprim | 774 (3.4) | 358 (4.0) | 1.17 (1.04-1.32) | 922 (4.3) | 402 (4.5) | 1.15 (1.03-1.29) | 62 (2.4) | 50 (3.2) | 1.32 (0.92-1.91) |
| Loop diuretics | 7,692 (33.4) | 4,075 (45.0) | 1.33 (1.29-1.37) | 7,700 (35.6) | 3,758 (42.5) | 1.22 (1.19-1.26) | 2,045 (80.7) | 1,326 (84.0) | 1.04 (1.01-1.07) |
| ^a^Adjusted for age and sex  Abbreviations: ACEis, angiotensin-converting enzyme inhibitors; ARBs, angiotensin-receptor II blockers; CI, confidence interval; CKD: Chronic kidney disease; eGFR, estimated Glomerular Filtration Rate; HK, hyperkalemia; NSAIDs, non-steroidal anti-inflammatory drugs; PR, prevalence ratio; RASi, renin angiotensin system inhibitors | | | | | | | | | |
