# Supplementary material for: Predictors for repeated hyperkalemia and potassium trajectories in high-risk patients — A population-based cohort study
Source: PLoS One. 2019 Jun 21;14(6):e0218739. doi: 10.1371/journal.pone.0218739 (PMC6588240; doi:10.1371/journal.pone.0218739)
Supplement: S6 Table — Restricted to patients surviving in the 6-month trajectory period. (DOCX) [file pone.0218739.s006.docx]

| **S6 Table**. **Prevalence of clinical predictors in patients with one and more than one hyperkalemia event during a 6-month trajectory period and corresponding prevalence ratios. Restricted to patients surviving in the 6-month trajectory period.** | | | | | | |
| --- | --- | --- | --- | --- | --- | --- |
|  | **RASi new-users** | | **Chronic kidney disease** | | **Chronic heart failure** | |
|  | **Dead or lost to follow-up, n (%)** | **PR^a^**  **(95% CI)** | **Dead or lost to follow-up, n (%)** | **PR^a^**  **(95% CI)** | **Dead or lost to follow-up, n (%)** | **PR^a^**  **(95% CI)** |
| **Total** | 8,993 (100) |  | 12,643 (100) |  | 1,697 (100) |  |
| **Median age (range)** | 78.1 (69.8-85.0) |  | 79.3 (71.0-85.8) |  | 78.9 (71.3-84.7) |  |
| **Female** | 4,352 (48.4) | 0.95 (0.92-0.97) | 6,248 (49.4) | 0.86 (0.84-0.88) | 725 (42.7) | 1.03 (0.95-1.12) |
| **K+ level (mmol/L)** |  |  |  |  |  |  |
| >5.0–5.5 | 6,434 (71.5) | 0.85 (0.84-0.86) | 8,763 (69.3) | 0.85 (0.84-0.86) | 1,220 (71.9) | 0.86 (0.84-0.89) |
| 5.6–6.0 | 1,557 (17.3) | 1.94 (1.82-2.07) | 2,331 (18.4) | 1.79 (1.68-1.90) | 300 (17.7) | 1.63 (1.38-1.91) |
| 6.1–6.5 | 540 (6.0) | 3.29 (2.86-3.79) | 852 (6.7) | 3.12 (2.72-3.58) | 94 (5.5) | 2.59 (1.78-3.77) |
| 6.6–7.0 | 264 (2.9) | 3.14 (2.47-3.99) | 373 (3.0) | 3.06 (2.47-3.78) | 44 (2.6) | 2.31 (1.28-4.18) |
| >7.0 | 198 (2.2) | 4.50 (3.47-5.83) | 324 (2.6) | 3.40 (2.71-4.27) | 39 (2.3) | 4.86 (2.44-9.66) |
| **eGFR groups (mL/min/1.73m2)** |  |  |  |  |  |  |
| Not measured | 104 (1.2) | 0.94 (0.80-1.11) | 70 (0.6) | 2.92 (2.06-4.14) | 112 (6.6) | 0.68 (0.54-0.85) |
| ≥60 | 1,616 (18.0) | 0.55 (0.53-0.58) | 106 (0.8) | 1.14 (0.89-1.46) | 122 (7.2) | 0.53 (0.45-0.64) |
| 45–59 | 1,902 (21.1) | 0.80 (0.77-0.83) | 3,596 (28.4) | 0.65 (0.63-0.67) | 235 (13.8) | 0.77 (0.68-0.88) |
| 30–44 | 2,469 (27.5) | 1.18 (1.13-1.22) | 4,227 (33.4) | 1.06 (1.02-1.09) | 430 (25.3) | 0.97 (0.89-1.07) |
| 15–29 | 2,154 (24.0) | 1.67 (1.59-1.76) | 3,374 (26.7) | 1.49 (1.42-1.56) | 556 (32.8) | 1.45 (1.29-1.62) |
| <15 | 657 (7.3) | 2.70 (2.42-3.01) | 1,142 (9.0) | 2.40 (2.18-2.65) | 187 (11.0) | 1.59 (1.24-2.04) |
| Dialysis | 91 (1.0) | 6.14 (5.04-7.49) | 128 (1.0) | 3.38 (2.71-4.21) | 55 (3.2) | 3.88 (2.54-5.91) |
| **Comorbidities** |  |  |  |  |  |  |
| Diabetes | 2,602 (28.9) | 1.17 (1.13-1.21) | 2,872 (22.7) | 1.24 (1.20-1.29) | 621 (36.6) | 1.20 (1.09-1.31) |
| CKD | 5,593 (62.2) | 1.24 (1.22-1.27) | N/A | N/A | 1,296 (76.4) | 1.17 (1.12-1.22) |
| Heart failure | 2,403 (26.7) | 1.40 (1.33-1.46) | 3,023 (23.9) | 1.33 (1.27-1.40) | N/A | N/A |
| IHD | 2,914 (32.4) | 1.06 (1.03-1.10) | 3,356 (26.5) | 1.12 (1.07-1.16) | 1,133 (66.8) | 0.95 (0.91-1.00) |
| **Comedication** |  |  |  |  |  |  |
| ACEis | 5,736 (63.8) | 1.04 (1.03-1.06) | 3,798 (30.0) | 1.19 (1.16-1.23) | 1,190 (70.1) | 1.05 (1.01-1.09) |
| ARBs | 2,468 (27.4) | 0.99 (0.96-1.02) | 1,827 (14.5) | 1.12 (1.07-1.17) | 387 (22.8) | 1.07 (0.96-1.20) |
| Spironolactone | 1,929 (21.5) | 1.49 (1.43-1.56) | 2,503 (19.8) | 1.26 (1.18-1.36) | 732 (43.1) | 1.09 (1.03-1.16) |
| Macrolides | 1,255 (14.0) | 1.14 (1.07-1.22) | 0 | N/A | 270 (15.9) | 1.27 (1.08-1.49) |
| ^a^Adjusted for age and sex  Abbreviations: ACEis, angiotensin-converting enzyme inhibitors; ARBs, angiotensin-receptor II blockers; CI, confidence interval; CKD: chronic kidney disease; eGFR, estimated glomerular filtration rate; IHD, ischemic heart disease; PR, prevalence ratio; RASi, renin angiotensin system inhibitors | | | | | | |
