# Supplementary material for: Predictors for repeated hyperkalemia and potassium trajectories in high-risk patients — A population-based cohort study
Source: PLoS One. 2019 Jun 21;14(6):e0218739. doi: 10.1371/journal.pone.0218739 (PMC6588240; doi:10.1371/journal.pone.0218739)
Supplement: S7 Table — (DOCX) [file pone.0218739.s007.docx]

| **S7 Table**. **Prevalence of clinical predictors in patients with one and more than one hyperkalemia events during a 6-month trajectory period and corresponding prevalence ratios, changing the definition from repeated to recurrent hyperkalemia.** | | | | | | | | | |
| --- | --- | --- | --- | --- | --- | --- | --- | --- | --- |
|  | **RASi new-users** | | | **Chronic kidney disease** | | | **Chronic heart failure** | | |
|  | **1 HK event, n (%)** | **≥2 HK events, n (%)** | **PR^a^**  **(95% CI)** | **1 HK event, n (%)** | **≥2 HK events, n (%)** | **PR^a^**  **(95% CI)** | **1 HK event, n (%)** | **≥2 HK events, n (%)** | **PR^a^**  **(95% CI)** |
| **Total** | 26,164 (100) | 9,384 (100) |  | 26,257 (100) | 10,655 (100) |  | 2,885 (100) | 1,801 (100) |  |
| **Median (range) potassium tests 6 months before** | 2.00 (1.00-5.00) | 4.00 (2.00-10.00) |  | 2.00 (1.00-6.00) | 4.00 (2.00-10.00) |  | 5.00 (1.00-11.00) | 8.00 (3.00-15.00) |  |
| **Median (range) potassium tests 6 months after** | 3.00 (2.00-6.00) | 12.00 (7.00-22.00) |  | 3.00 (2.00-6.00) | 14.00 (8.00-24.00) |  | 4.00 (2.00-8.00) | 13.00 (8.00-23.00) |  |
| **Females** | 12,304 (47.0) | 4,132 (44.0) | 0.93 (0.90-0.95) | 14,209 (54.1) | 4,694 (44.1) | 0.83 (0.81-0.85) | 1,085 (37.6) | 679 (37.7) | 1.00 (0.93-1.08) |
| **Median age (range)** | 72.60 (62.80-81.10) | 74.21 (64.95-81.26) |  | 76.66 (67.53-83.81) | 74.92 (65.89-81.81) |  | 75.18 (67.17-82.22) | 75.65 (68.08-81.69) |  |
| **First-time K+ level (mmol/L)** |  |  |  |  |  |  |  |  |  |
| >5.0–5.5 | 22,759 (87.0) | 7,221 (77.0) | 0.88 (0.87-0.90) | 22,147 (84.3) | 7,901 (74.2) | 0.88 (0.87-0.89) | 2,406 (83.4) | 1,385 (76.9) | 0.92 (0.89-0.95) |
| 5.6–6.0 | 2,467 (9.4) | 1,481 (15.8) | 1.67 (1.57-1.77) | 2,884 (11.0) | 1,768 (16.6) | 1.52 (1.43-1.60) | 343 (11.9) | 287 (15.9) | 1.34 (1.16-1.55) |
| 6.1–6.5 | 548 (2.1) | 414 (4.4) | 2.10 (1.85-2.38) | 683 (2.6) | 555 (5.2) | 1.98 (1.77-2.21) | 72 (2.5) | 74 (4.1) | 1.64 (1.20-2.26) |
| 6.6–7.0 | 223 (0.9) | 156 (1.7) | 1.94 (1.58-2.37) | 295 (1.1) | 234 (2.2) | 1.90 (1.60-2.25) | 34 (1.2) | 28 (1.6) | 1.32 (0.80-2.17) |
| >7.0 | 167 (0.6) | 112 (1.2) | 1.89 (1.49-2.39) | 248 (0.9) | 197 (1.8) | 1.93 (1.60-2.32) | 30 (1.0) | 27 (1.5) | 1.44 (0.86-2.40) |
| **eGFR groups (mL/min/1.73m2)** |  |  |  |  |  |  |  |  |  |
| Not measured | 487 (1.9) | 121 (1.3) | 0.69 (0.57-0.85) | 72 (0.3) | 88 (0.8) | 2.50 (1.84-3.41) | 241 (8.4) | 98 (5.4) | 0.65 (0.52-0.82) |
| ≥60 | 8,104 (31.0) | 1,501 (16.0) | 0.52 (0.50-0.55) | 180 (0.7) | 125 (1.2) | 1.24 (0.98-1.58) | 381 (13.2) | 137 (7.6) | 0.56 (0.47-0.67) |
| 45–59 | 7,035 (26.9) | 1,998 (21.3) | 0.79 (0.76-0.82) | 11,343 (43.2) | 2,951 (27.7) | 0.63 (0.61-0.65) | 596 (20.7) | 256 (14.2) | 0.69 (0.60-0.79) |
| 30–44 | 6,068 (23.2) | 2,554 (27.2) | 1.15 (1.11-1.20) | 8,674 (33.0) | 3,519 (33.0) | 1.03 (1.00-1.07) | 825 (28.6) | 504 (28.0) | 0.98 (0.89-1.08) |
| 15–29 | 3,484 (13.3) | 2,088 (22.3) | 1.65 (1.57-1.73) | 4,677 (17.8) | 2,646 (24.8) | 1.44 (1.38-1.50) | 613 (21.2) | 536 (29.8) | 1.41 (1.27-1.55) |
| <15 | 821 (3.1) | 768 (8.2) | 2.62 (2.38-2.89) | 1,137 (4.3) | 1,071 (10.1) | 2.23 (2.05-2.41) | 181 (6.3) | 168 (9.3) | 1.49 (1.22-1.82) |
| Dialysis | 165 (0.6) | 354 (3.8) | 6.03 (5.03-7.24) | 174 (0.7) | 255 (2.4) | 3.01 (2.48-3.66) | 48 (1.7) | 102 (5.7) | 3.34 (2.39-4.67) |
| **Comorbidities** |  |  |  |  |  |  |  |  |  |
| Diabetes | 7,799 (29.8) | 3,151 (33.6) | 1.13 (1.10-1.17) | 6,128 (23.3) | 3,046 (28.6) | 1.18 (1.14-1.22) | 883 (30.6) | 673 (37.4) | 1.22 (1.13-1.32) |
| Chronic kidney disease | 13,519 (51.7) | 6,139 (65.4) | 1.25 (1.23-1.28) | 22,476 (85.6) | 9,131 (85.7) | 1.01 (1.00-1.02) | 1,892 (65.6) | 1,382 (76.7) | 1.17 (1.13-1.21) |
| Heart failure | 4,501 (17.2) | 2,418 (25.8) | 1.45 (1.39-1.52) | 4,628 (17.6) | 2,498 (23.4) | 1.35 (1.29-1.41) | 2,879 (99.8) | 1,795 (99.7) | 1.00 (1.00-1.00) |
| Ischemic heart disease | 6,947 (26.6) | 2,965 (31.6) | 1.15 (1.11-1.20) | 6,272 (23.9) | 2,979 (28.0) | 1.15 (1.10-1.19) | 1,876 (65.0) | 1,160 (64.4) | 0.99 (0.95-1.03) |
| Hypertension | 22,946 (87.7) | 8,620 (91.9) | 1.04 (1.04-1.05) | 17,945 (68.3) | 7,765 (72.9) | 1.08 (1.06-1.09) | 2,878 (99.8) | 1,799 (99.9) | 1.00 (1.00-1.00) |
| Atrial fibrillation or flutter | 4,459 (17.0) | 2,182 (23.3) | 1.32 (1.26-1.38) | 4,966 (18.9) | 2,339 (22.0) | 1.20 (1.15-1.26) | 1,317 (45.6) | 834 (46.3) | 1.02 (0.95-1.08) |
| Valvular heart disease | 2,161 (8.3) | 1,137 (12.1) | 1.44 (1.35-1.54) | 2,127 (8.1) | 1,130 (10.6) | 1.35 (1.26-1.45) | 619 (21.5) | 428 (23.8) | 1.11 (1.00-1.24) |
| Cardiomyopathy | 811 (3.1) | 414 (4.4) | 1.43 (1.27-1.61) | 686 (2.6) | 377 (3.5) | 1.20 (1.06-1.36) | 482 (16.7) | 283 (15.7) | 0.93 (0.81-1.06) |
| Peripheral vascular disease | 2,893 (11.1) | 1,413 (15.1) | 1.34 (1.26-1.42) | 3,049 (11.6) | 1,559 (14.6) | 1.25 (1.18-1.33) | 507 (17.6) | 387 (21.5) | 1.22 (1.09-1.38) |
| Cerebrovascular disease | 4,595 (17.6) | 1,770 (18.9) | 1.05 (1.00-1.10) | 4,867 (18.5) | 1,857 (17.4) | 0.95 (0.91-1.00) | 542 (18.8) | 354 (19.7) | 1.05 (0.93-1.18) |
| Dementia | 461 (1.8) | 120 (1.3) | 0.71 (0.58-0.86) | 666 (2.5) | 150 (1.4) | 0.63 (0.53-0.75) | 32 (1.1) | 18 (1.0) | 0.91 (0.51-1.61) |
| Chronic pulmonary disease | 3,887 (14.9) | 1,825 (19.4) | 1.30 (1.23-1.37) | 4,393 (16.7) | 2,202 (20.7) | 1.24 (1.18-1.30) | 633 (21.9) | 471 (26.2) | 1.19 (1.07-1.32) |
| Connective tissue disease | 1,299 (5.0) | 588 (6.3) | 1.28 (1.17-1.41) | 1,486 (5.7) | 661 (6.2) | 1.16 (1.06-1.26) | 197 (6.8) | 122 (6.8) | 0.99 (0.80-1.23) |
| Peptic ulcer disease | 2,180 (8.3) | 994 (10.6) | 1.25 (1.17-1.35) | 2,645 (10.1) | 1,136 (10.7) | 1.07 (1.00-1.14) | 312 (10.8) | 206 (11.4) | 1.06 (0.90-1.25) |
| Any cancer | 4,149 (15.9) | 2,028 (21.6) | 1.34 (1.28-1.41) | 5,378 (20.5) | 2,665 (25.0) | 1.22 (1.18-1.28) | 414 (14.4) | 298 (16.5) | 1.15 (1.01-1.32) |
| Alcoholism-related disorders | 2,309 (8.8) | 967 (10.3) | 1.18 (1.10-1.27) | 2,291 (8.7) | 1,236 (11.6) | 1.18 (1.10-1.26) | 308 (10.7) | 199 (11.0) | 1.02 (0.86-1.21) |
| Obesity | 1,932 (7.4) | 842 (9.0) | 1.26 (1.16-1.36) | 1,624 (6.2) | 830 (7.8) | 1.21 (1.11-1.31) | 294 (10.2) | 196 (10.9) | 1.05 (0.89-1.25) |
| **Comedication** |  |  |  |  |  |  |  |  |  |
| ACEis | 17,404 (66.5) | 6,383 (68.0) | 1.02 (1.00-1.04) | 9,012 (34.3) | 4,064 (38.1) | 1.10 (1.06-1.13) | 2,102 (72.9) | 1,353 (75.1) | 1.03 (1.00-1.07) |
| ARBs | 7,958 (30.4) | 2,801 (29.8) | 0.99 (0.95-1.02) | 4,523 (17.2) | 2,011 (18.9) | 1.10 (1.05-1.15) | 664 (23.0) | 440 (24.4) | 1.06 (0.96-1.18) |
| Spironolactone | 4,334 (16.6) | 2,177 (23.2) | 1.39 (1.33-1.45) | 4,371 (16.6) | 2,254 (21.2) | 1.30 (1.24-1.36) | 1,332 (46.2) | 876 (48.6) | 1.05 (0.99-1.12) |
| Macrolides | 2,765 (10.6) | 1,197 (12.8) | 1.22 (1.14-1.30) | 0 (0.0) | 0 (0.0) | N/A | 351 (12.2) | 310 (17.2) | 1.41 (1.23-1.63) |
| Beta blockers | 11,482 (43.9) | 4,687 (49.9) | 1.13 (1.10-1.16) | 9,321 (35.5) | 4,123 (38.7) | 1.09 (1.06-1.12) | 2,508 (86.9) | 1,614 (89.6) | 1.03 (1.01-1.05) |
| Azoles | 796 (3.0 | 371 (4.0) | 1.34 (1.19-1.51) | 929 (3.5) | 421 (4.0) | 1.15 (1.02-1.28) | 87 (3.0) | 71 (3.9) | 1.30 (0.96-1.77) |
| Digoxin | 2,306 (8.8) | 1,149 (12.2) | 1.35 (1.26-1.44) | 2,487 (9.5) | 1,182 (11.1) | 1.26 (1.18-1.34) | 791 (27.4) | 479 (26.6) | 0.97 (0.88-1.07) |
| NSAIDs | 7,648 (29.2) | 2,817 (30.0) | 1.03 (1.00-1.07) | 7,090 (27.0) | 2,947 (27.7) | 1.03 (1.00-1.07) | 600 (20.8) | 421 (23.4) | 1.12 (1.01-1.25) |
| Potassium supplements | 7,113 (27.2) | 3,322 (35.4) | 1.28 (1.24-1.33) | 7,675 (29.2) | 3,487 (32.7) | 1.19 (1.16-1.23) | 1,599 (55.4) | 1,045 (58.0) | 1.05 (1.00-1.10) |
| Trimethoprim | 841 (3.2) | 389 (4.1) | 1.28 (1.14-1.44) | 1,103 (4.2) | 427 (4.0) | 1.06 (0.95-1.18) | 85 (2.9) | 57 (3.2) | 1.09 (0.78-1.51) |
| Loop diuretics | 8,651 (33.1) | 4,599 (49.0) | 1.46 (1.42-1.50) | 9,155 (34.9) | 4,632 (43.5) | 1.30 (1.26-1.33) | 2,322 (80.5) | 1,543 (85.7) | 1.06 (1.04-1.09) |
| ^a^Adjusted for age and sex  Abbreviations: ACEis, angiotensin-converting enzyme inhibitors; ARBs, angiotensin-receptor II blockers; CI, confidence interval; CKD: Chronic kidney disease; eGFR, estimated Glomerular Filtration Rate; HK, hyperkalemia; NSAIDs, non-steroidal anti-inflammatory drugs; PR, prevalence ratio; RASi, renin angiotensin system inhibitors | | | | | | | | | |
