# Supplementary material for: Predictors for repeated hyperkalemia and potassium trajectories in high-risk patients — A population-based cohort study
Source: PLoS One. 2019 Jun 21;14(6):e0218739. doi: 10.1371/journal.pone.0218739 (PMC6588240; doi:10.1371/journal.pone.0218739)
Supplement: S8 Table — (DOCX) [file pone.0218739.s008.docx]

| **S8 Table**. **Prevalence of clinical predictors in patients with one and more than one hyperkalemia events during a 6-month trajectory period and corresponding prevalence ratios, requiring at least two potassium tests during the trajectory period.** | | | | | | | | | |
| --- | --- | --- | --- | --- | --- | --- | --- | --- | --- |
|  | **RASi new-users** | | | **Chronic kidney disease** | | | **Chronic heart failure** | | |
|  | **1 HK event, n (%)** | **≥2 HK events, n (%)** | **PR^a^**  **(95% CI)** | **1 HK event, n (%)** | **≥2 HK events, n (%)** | **PR^a^**  **(95% CI)** | **1 HK event, n (%)** | **≥2 HK events, n (%)** | **PR^a^**  **(95% CI)** |
| **Total** | 19,594 (100) | 14,637 (100) |  | 19,774 (100) | 17,588 (100) |  | 2,198 (100) | 2,436 (100) |  |
| **Median (range) potassium tests 6 months before** | 2.00 (1.00-6.00) | 4.00 (1.00-9.00) |  | 2.00 (1.00-6.00) | 4.00 (1.00-9.00) |  | 6.00 (2.00-11.00) | 8.00 (3.00-15.00) |  |
| **Median (range) potassium tests 6 months after** | 4.00 (2.00-7.00) | 8.00 (4.00-16.00) |  | 4.00 (2.00-8.00) | 9.00 (5.00-18.00) |  | 5.00 (3.00-10.00) | 10.00 (5.00-19.00) |  |
| **Females** | 9,205 (47.0) | 6,634 (45.3) | 0.94 (0.92-0.96) | 10,503 (53.1) | 8,145 (46.3) | 0.88 (0.86-0.89) | 828 (37.7) | 936 (38.4) | 1.00 (0.93-1.08) |
| **Median age (range)** | 72.74 (63.12-80.99) | 75.13 (65.76-82.18) |  | 76.35 (67.43-83.44) | 75.90 (66.71-82.79) |  | 75.17 (67.20-81.91) | 76.24 (68.45-82.44) |  |
| **First-time K+ level (mmol/L)** |  |  |  |  |  |  |  |  |  |
| >5.0–5.5 | 17,535 (89.5) | 11,209 (76.6) | 0.86 (0.85-0.87) | 16,910 (85.5) | 12,537 (71.3) | 0.83 (0.82-0.84) | 1,889 (85.9) | 1,824 (74.9) | 0.87 (0.85-0.90) |
| 5.6–6.0 | 1,656 (8.5) | 2,373 (16.2) | 1.91 (1.80-2.02) | 2,080 (10.5) | 3,133 (17.8) | 1.70 (1.61-1.79) | 249 (11.3) | 429 (17.6) | 1.55 (1.34-1.79) |
| 6.1–6.5 | 282 (1.4) | 663 (4.5) | 3.09 (2.69-3.55) | 454 (2.3) | 1,062 (6.0) | 2.61 (2.34-2.90) | 44 (2.0) | 121 (5.0) | 2.47 (1.76-3.47) |
| 6.6–7.0 | 79 (0.4) | 228 (1.6) | 3.76 (2.91-4.84) | 183 (0.9) | 448 (2.5) | 2.72 (2.29-3.23) | 12 (0.5) | 27 (1.1) | 2.03 (1.03-3.99) |
| >7.0 | 42 (0.2) | 164 (1.1) | 5.22 (3.73-7.29) | 147 (0.7) | 408 (2.3) | 3.14 (2.60-3.79) | 4 (0.2) | 35 (1.4) | 8.13 (2.89-22.84) |
| **eGFR groups (mL/min/1.73m2)** |  |  |  |  |  |  |  |  |  |
| Not measured | 306 (1.6) | 246 (1.7) | 1.07 (0.91-1.27) | 53 (0.3) | 141 (0.8) | 2.77 (2.02-3.80) | 22 (1.0) | 15 (0.6) | 0.60 (0.31-1.16) |
| ≥60 | 5,708 (29.1) | 2,336 (16.0) | 0.57 (0.55-0.60) | 139 (0.7) | 197 (1.1) | 1.34 (1.07-1.67) | 305 (13.9) | 172 (7.1) | 0.53 (0.45-0.63) |
| 45–59 | 5,314 (27.1) | 3,063 (20.9) | 0.77 (0.74-0.80) | 8,276 (41.9) | 5,148 (29.3) | 0.69 (0.67-0.71) | 478 (21.7) | 390 (16.0) | 0.75 (0.66-0.84) |
| 30–44 | 4,701 (24.0) | 4,114 (28.1) | 1.12 (1.08-1.16) | 6,647 (33.6) | 5,856 (33.3) | 1.00 (0.97-1.03) | 684 (31.1) | 723 (29.7) | 0.94 (0.86-1.03) |
| 15–29 | 2,752 (14.0) | 3,324 (22.7) | 1.56 (1.49-1.63) | 3,644 (18.4) | 4,317 (24.5) | 1.35 (1.30-1.40) | 526 (23.9) | 770 (31.6) | 1.29 (1.18-1.42) |
| <15 | 667 (3.4) | 1,102 (7.5) | 2.24 (2.04-2.46) | 875 (4.4) | 1,597 (9.1) | 2.01 (1.85-2.17) | 141 (6.4) | 244 (10.0) | 1.55 (1.27-1.89) |
| Dialysis | 146 (0.7) | 452 (3.1) | 4.35 (3.62-5.23) | 140 (0.7) | 332 (1.9) | 2.42 (1.99-2.95) | 42 (1.9) | 122 (5.0) | 2.70 (1.91-3.82) |
| **Comorbidities** |  |  |  |  |  |  |  |  |  |
| Diabetes | 6,163 (31.5) | 4,924 (33.6) | 1.09 (1.05-1.12) | 4,941 (25.0) | 4,995 (28.4) | 1.12 (1.08-1.15) | 686 (31.2) | 884 (36.3) | 1.17 (1.08-1.27) |
| Chronic kidney disease | 10,472 (53.4) | 9,579 (65.4) | 1.19 (1.17-1.21) | 17,148 (86.7) | 14,791 (84.1) | 0.97 (0.97-0.98) | 1,466 (66.7) | 1,857 (76.2) | 1.13 (1.09-1.17) |
| Heart failure | 3,616 (18.5) | 3,626 (24.8) | 1.29 (1.23-1.34) | 3,648 (18.4) | 4,009 (22.8) | 1.23 (1.18-1.28) | 2,196 (99.9) | 2,432 (99.8) | 1.00 (1.00-1.00) |
| Ischemic heart disease | 5,451 (27.8) | 4,412 (30.1) | 1.04 (1.01-1.08) | 4,978 (25.2) | 4,750 (27.0) | 1.05 (1.01-1.08) | 1,428 (65.0) | 1,550 (63.6) | 0.98 (0.93-1.02) |
| Hypertension | 17,413 (88.9) | 13,394 (91.5) | 1.02 (1.02-1.03) | 14,001 (70.8) | 12,933 (73.5) | 1.04 (1.03-1.05) | 2,191 (99.7) | 2,432 (99.8) | 1.00 (1.00-1.00) |
| Atrial fibrillation or flutter | 3,573 (18.2) | 3,255 (22.2) | 1.15 (1.10-1.20) | 3,923 (19.8) | 3,793 (21.6) | 1.09 (1.05-1.13) | 1,028 (46.8) | 1,179 (48.4) | 1.02 (0.96-1.08) |
| Valvular heart disease | 1,714 (8.7) | 1,578 (10.8) | 1.20 (1.12-1.28) | 1,718 (8.7) | 1,763 (10.0) | 1.16 (1.09-1.24) | 488 (22.2) | 571 (23.4) | 1.04 (0.94-1.16) |
| Cardiomyopathy | 680 (3.5) | 569 (3.9) | 1.16 (1.04-1.29) | 584 (3.0) | 560 (3.2) | 1.01 (0.90-1.14) | 388 (17.7) | 363 (14.9) | 0.87 (0.77-0.99) |
| Peripheral vascular disease | 2,264 (11.6) | 2,051 (14.0) | 1.18 (1.11-1.25) | 2,383 (12.1) | 2,408 (13.7) | 1.12 (1.06-1.18) | 427 (19.4) | 528 (21.7) | 1.11 (0.99-1.25) |
| Cerebrovascular disease | 3,465 (17.7) | 2,773 (18.9) | 1.03 (0.98-1.08) | 3,740 (18.9) | 3,137 (17.8) | 0.94 (0.90-0.98) | 440 (20.0) | 518 (21.3) | 1.05 (0.94-1.18) |
| Dementia | 328 (1.7) | 215 (1.5) | 0.80 (0.68-0.95) | 438 (2.2) | 299 (1.7) | 0.80 (0.69-0.92) | 27 (1.2) | 32 (1.3) | 1.03 (0.62-1.71) |
| Chronic pulmonary disease | 3,064 (15.6) | 2,613 (17.9) | 1.12 (1.07-1.18) | 3,444 (17.4) | 3,433 (19.5) | 1.12 (1.07-1.17) | 531 (24.2) | 625 (25.7) | 1.06 (0.96-1.17) |
| Connective tissue disease | 1,057 (5.4) | 832 (5.7) | 1.06 (0.97-1.16) | 1,203 (6.1) | 996 (5.7) | 0.96 (0.89-1.05) | 164 (7.5) | 154 (6.3) | 0.84 (0.68-1.04) |
| Peptic ulcer disease | 1,705 (8.7) | 1,483 (10.1) | 1.14 (1.06-1.22) | 2,034 (10.3) | 1,824 (10.4) | 1.01 (0.95-1.07) | 249 (11.3) | 310 (12.7) | 1.11 (0.95-1.30) |
| Any cancer | 3,156 (16.1) | 3,036 (20.7) | 1.25 (1.20-1.31) | 4,098 (20.7) | 4,321 (24.6) | 1.18 (1.14-1.23) | 346 (15.7) | 436 (17.9) | 1.12 (0.99-1.28) |
| Alcoholism-related disorders | 1,789 (9.1) | 1,373 (9.4) | 1.07 (1.00-1.14) | 1,825 (9.2) | 1,868 (10.6) | 1.09 (1.02-1.15) | 243 (11.1) | 248 (10.2) | 0.95 (0.80-1.12) |
| Obesity | 1,523 (7.8) | 1,302 (8.9) | 1.20 (1.12-1.29) | 1,331 (6.7) | 1,362 (7.7) | 1.14 (1.06-1.23) | 244 (11.1) | 258 (10.6) | 0.98 (0.83-1.16) |
| **Comedication** |  |  |  |  |  |  |  |  |  |
| ACEis | 13,170 (67.2) | 10,077 (68.8) | 1.02 (1.01-1.04) | 7,127 (36.0) | 6,869 (39.1) | 1.07 (1.05-1.10) | 1,723 (78.4) | 1,956 (80.3) | 1.03 (1.00-1.06) |
| ARBs | 5,956 (30.4) | 4,421 (30.2) | 1.00 (0.97-1.03) | 3,606 (18.2) | 3,347 (19.0) | 1.05 (1.00-1.09) | 554 (25.2) | 626 (25.7) | 1.02 (0.93-1.13) |
| Spironolactone | 3,577 (18.3) | 3,648 (24.9) | 1.34 (1.29-1.40) | 3,496 (17.7) | 3,870 (22.0) | 1.25 (1.20-1.31) | 1,139 (51.8) | 1,339 (55.0) | 1.07 (1.01-1.12) |
| Macrolides | 2,154 (11.0) | 1,734 (11.8) | 1.09 (1.02-1.15) | 0 (0.0) | 0 (0.0) | N/A | 295 (13.4) | 412 (16.9) | 1.27 (1.10-1.45) |
| Beta blockers | 8,906 (45.5) | 7,197 (49.2) | 1.07 (1.05-1.10) | 7,355 (37.2) | 6,837 (38.9) | 1.04 (1.02-1.07) | 2,059 (93.7) | 2,296 (94.3) | 1.01 (0.99-1.02) |
| Azoles | 603 (3.1) | 552 (3.8) | 1.27 (1.13-1.42) | 709 (3.6) | 705 (4.0) | 1.15 (1.04-1.28) | 64 (2.9) | 93 (3.8) | 1.31 (0.96-1.80) |
| Digoxin | 1,854 (9.5) | 1,773 (12.1) | 1.20 (1.13-1.27) | 1,954 (9.9) | 1,976 (11.2) | 1.16 (1.09-1.23) | 658 (29.9) | 695 (28.5) | 0.94 (0.86-1.03) |
| NSAIDs | 5,849 (29.9) | 4,421 (30.2) | 1.02 (0.99-1.05) | 5,476 (27.7) | 4,925 (28.0) | 1.02 (0.99-1.05) | 490 (22.3) | 593 (24.3) | 1.09 (0.98-1.21) |
| Potassium supplements | 5,720 (29.2) | 5,113 (34.9) | 1.15 (1.12-1.19) | 6,041 (30.6) | 5,830 (33.1) | 1.11 (1.08-1.14) | 1,331 (60.6) | 1,477 (60.6) | 0.99 (0.95-1.04) |
| Trimethoprim | 677 (3.5) | 619 (4.2) | 1.17 (1.05-1.30) | 857 (4.3) | 769 (4.4) | 1.05 (0.96-1.16) | 67 (3.0) | 91 (3.7) | 1.19 (0.87-1.62) |
| Loop diuretics | 6,977 (35.6) | 7,005 (47.9) | 1.30 (1.27-1.34) | 7,167 (36.2) | 7,622 (43.3) | 1.21 (1.18-1.24) | 1,824 (83.0) | 2,119 (87.0) | 1.05 (1.02-1.07) |
| ^a^Adjusted for age and sex  Abbreviations: ACEis, angiotensin-converting enzyme inhibitors; ARBs, angiotensin-receptor II blockers; CI, confidence interval; CKD: Chronic kidney disease; eGFR, estimated Glomerular Filtration Rate; HK, hyperkalemia; NSAIDs, non-steroidal anti-inflammatory drugs; PR, prevalence ratio; RASi, renin angiotensin system inhibitors | | | | | | | | | |
