# Supplementary material for: Predictors for repeated hyperkalemia and potassium trajectories in high-risk patients — A population-based cohort study
Source: PLoS One. 2019 Jun 21;14(6):e0218739. doi: 10.1371/journal.pone.0218739 (PMC6588240; doi:10.1371/journal.pone.0218739)
Supplement: S1 Fig — A. The overall study design, illustrating the 6 month trajectory period following hyperkalemia. B. One patient enrolled in the RASi new user cohort and subsequently in the CKD cohort. C. One patient enrolled in the CHF cohort at the time of fulfilling all the CHF criteria: echocardiography, hospitalization with CHF, redeemed prescriptions of ACEi and beta blocker. (DOCX) [file pone.0218739.s009.docx]

**S1 Fig. Time frame of the cohort study with examples of the different cohort entries.**

A. The overall study design, illustrating the 6 month trajectory period following hyperkalemia.

B. One patient enrolled in the RASi new user cohort and subsequently in the CKD cohort.

C. One patient enrolled in the CHF cohort at the time of fulfilling all the CHF criteria: echocardiography, hospitalization with CHF, redeemed prescriptions of ACEi and beta blocker.

Abbreviations: ACEi, angiotensin-converting enzyme inhibitor; CHF, chronic heart failure; CKD: chronic kidney disease; eGFR, estimated glomerular filtration rate; RASi, renin angiotensin system inhibitors
