# Supplementary material for: Predictors for repeated hyperkalemia and potassium trajectories in high-risk patients — A population-based cohort study
Source: PLoS One. 2019 Jun 21;14(6):e0218739. doi: 10.1371/journal.pone.0218739 (PMC6588240; doi:10.1371/journal.pone.0218739)
Supplement: S2 Fig — (DOCX) [file pone.0218739.s010.docx]

**S2 Fig**. **Median potassium levels (mmol/L) in the three patient cohorts before and after a first hyperkalemia event, restricted to measurements at general practitioners.**

**
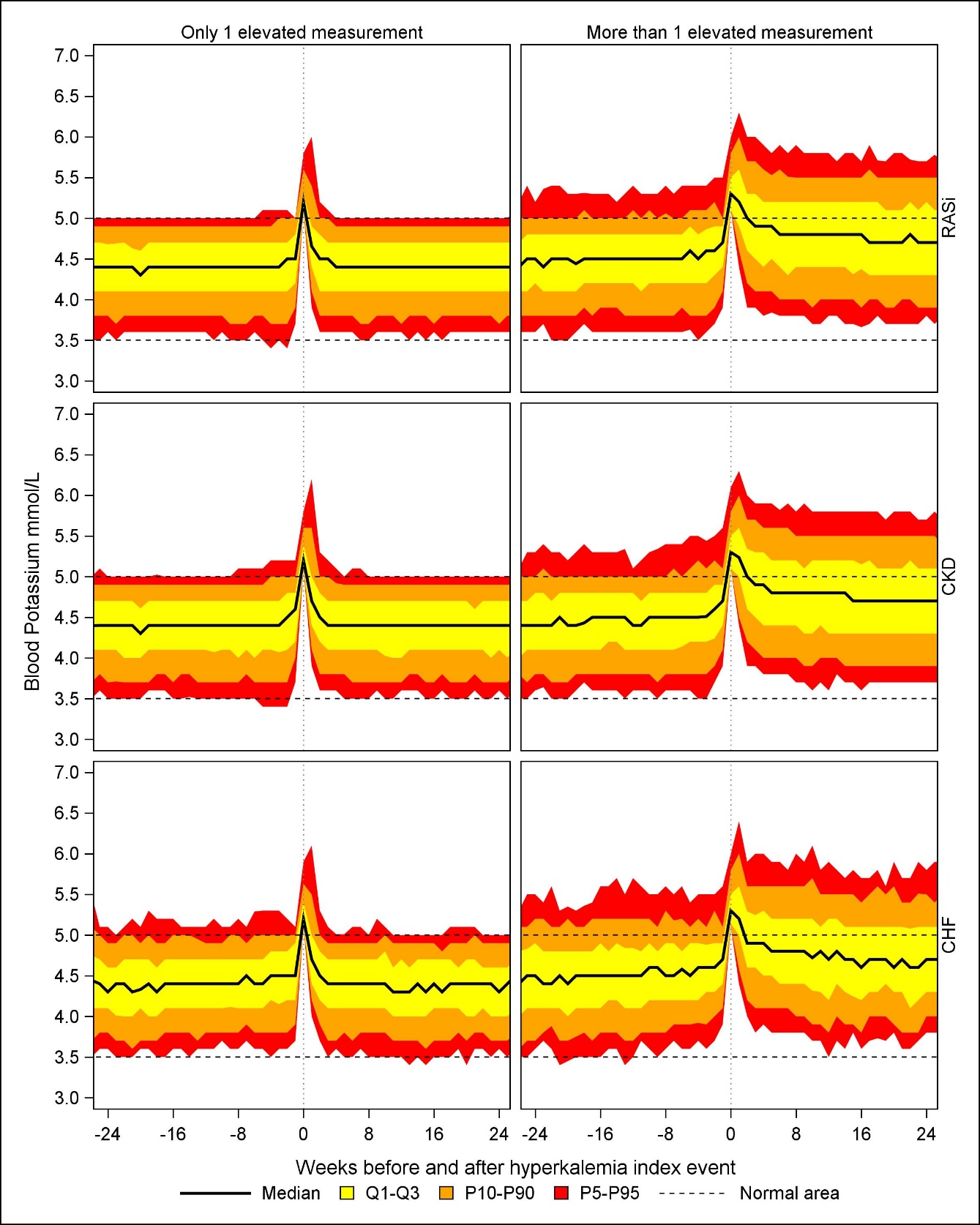
**

Abbreviations: CHF, chronic heart failure; CKD, chronic kidney disease; RASi, renin angiotensin system inhibitors
