# Supplementary material for: Predictors for repeated hyperkalemia and potassium trajectories in high-risk patients — A population-based cohort study
Source: PLoS One. 2019 Jun 21;14(6):e0218739. doi: 10.1371/journal.pone.0218739 (PMC6588240; doi:10.1371/journal.pone.0218739)
Supplement: S3 Fig — (DOCX) [file pone.0218739.s011.docx]

**S3 Fig**. **All potassium test results for 50 randomly sampled individuals in three cohorts before and after first hyperkalemia event, by patients with one and more than one hyperkalemia event, according to measurements at general practitioners.**


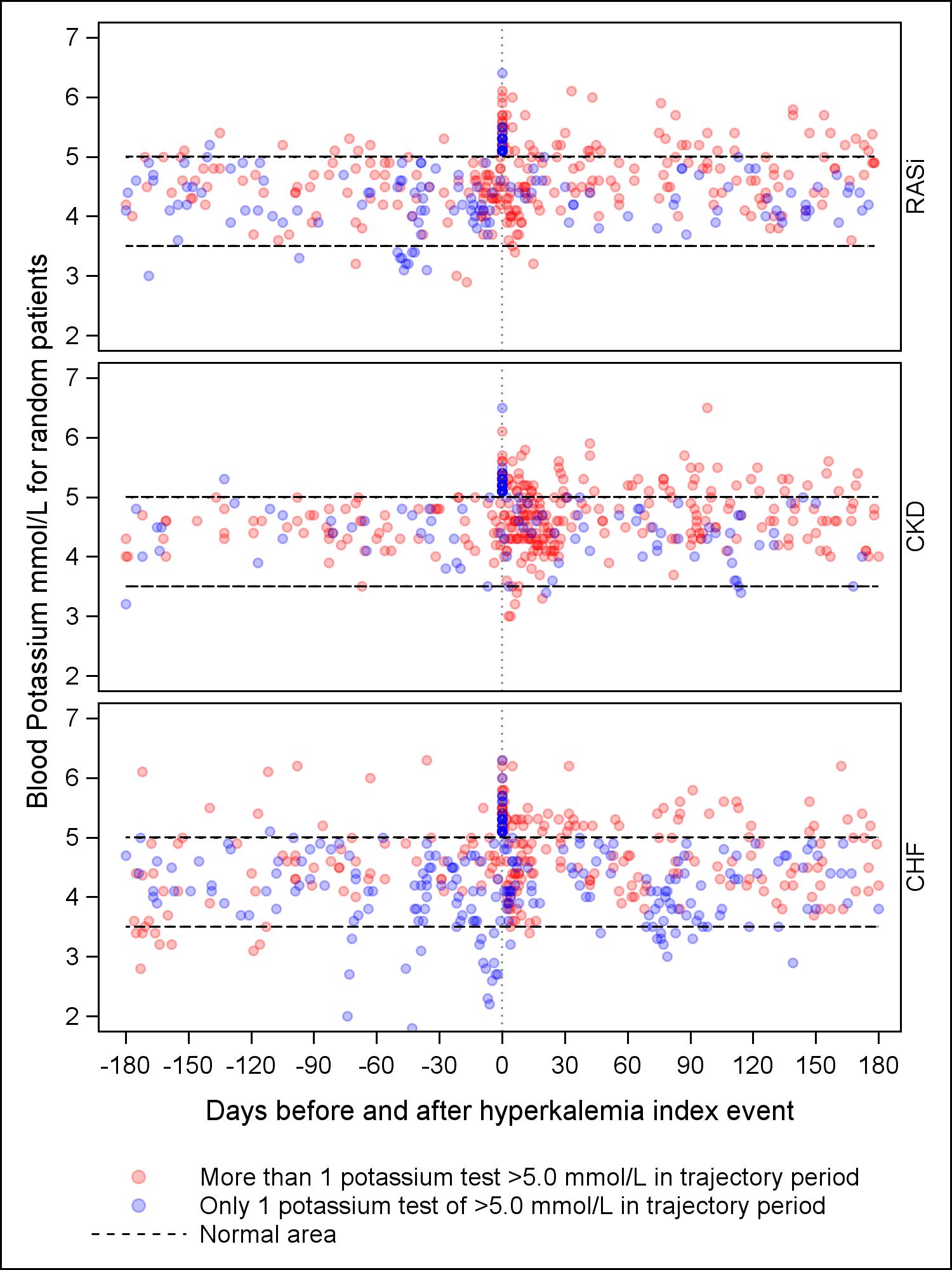


Abbreviations: CHF, chronic heart failure; CKD, chronic kidney disease; RASi, renin angiotensin system inhibitors
