# Supplementary material for: Predictors for repeated hyperkalemia and potassium trajectories in high-risk patients — A population-based cohort study
Source: PLoS One. 2019 Jun 21;14(6):e0218739. doi: 10.1371/journal.pone.0218739 (PMC6588240; doi:10.1371/journal.pone.0218739)
Supplement: S4 Fig — (DOCX) [file pone.0218739.s012.docx]

**S4 Fig. Proportion of potassium level tests results above 5.0 mmol/L in relation to total number of potassium test at the same day, before and after the first index hyperkalemia event, according to measurements at general practitioners.**


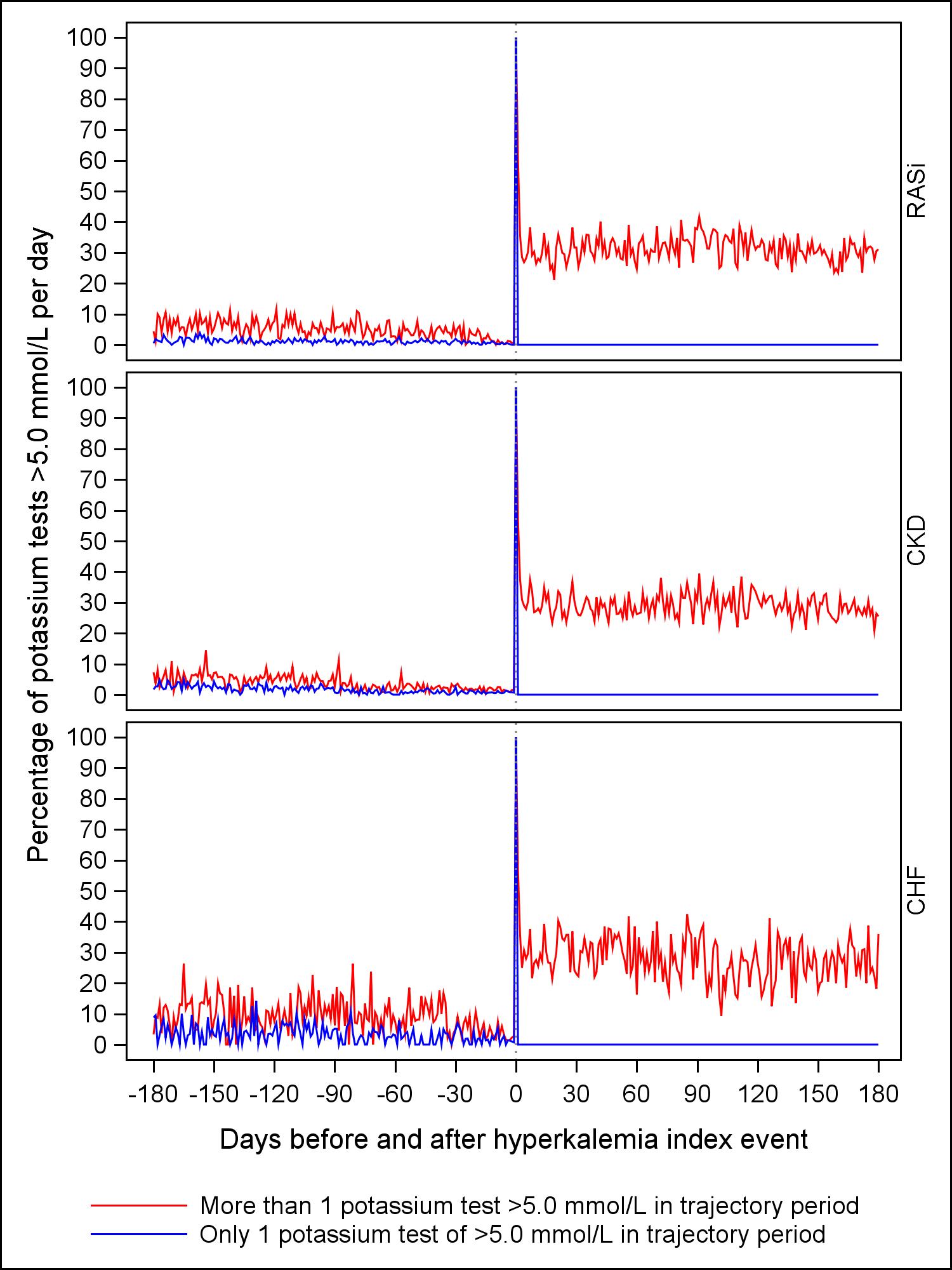


Abbreviations: CHF, chronic heart failure; CKD, chronic kidney disease; RASi, renin angiotensin system inhibitors
