# Supplementary material for: Predictors for repeated hyperkalemia and potassium trajectories in high-risk patients — A population-based cohort study
Source: PLoS One. 2019 Jun 21;14(6):e0218739. doi: 10.1371/journal.pone.0218739 (PMC6588240; doi:10.1371/journal.pone.0218739)
Supplement: S5 Fig — (DOCX) [file pone.0218739.s013.docx]

**S5 Fig. Proportion of patients hospitalized or covered with selected prescription drugs of interest before and after the first index hyperkalemia event, by patients with one and more than one hyperkalemia event, according to measurements at general practitioners.**


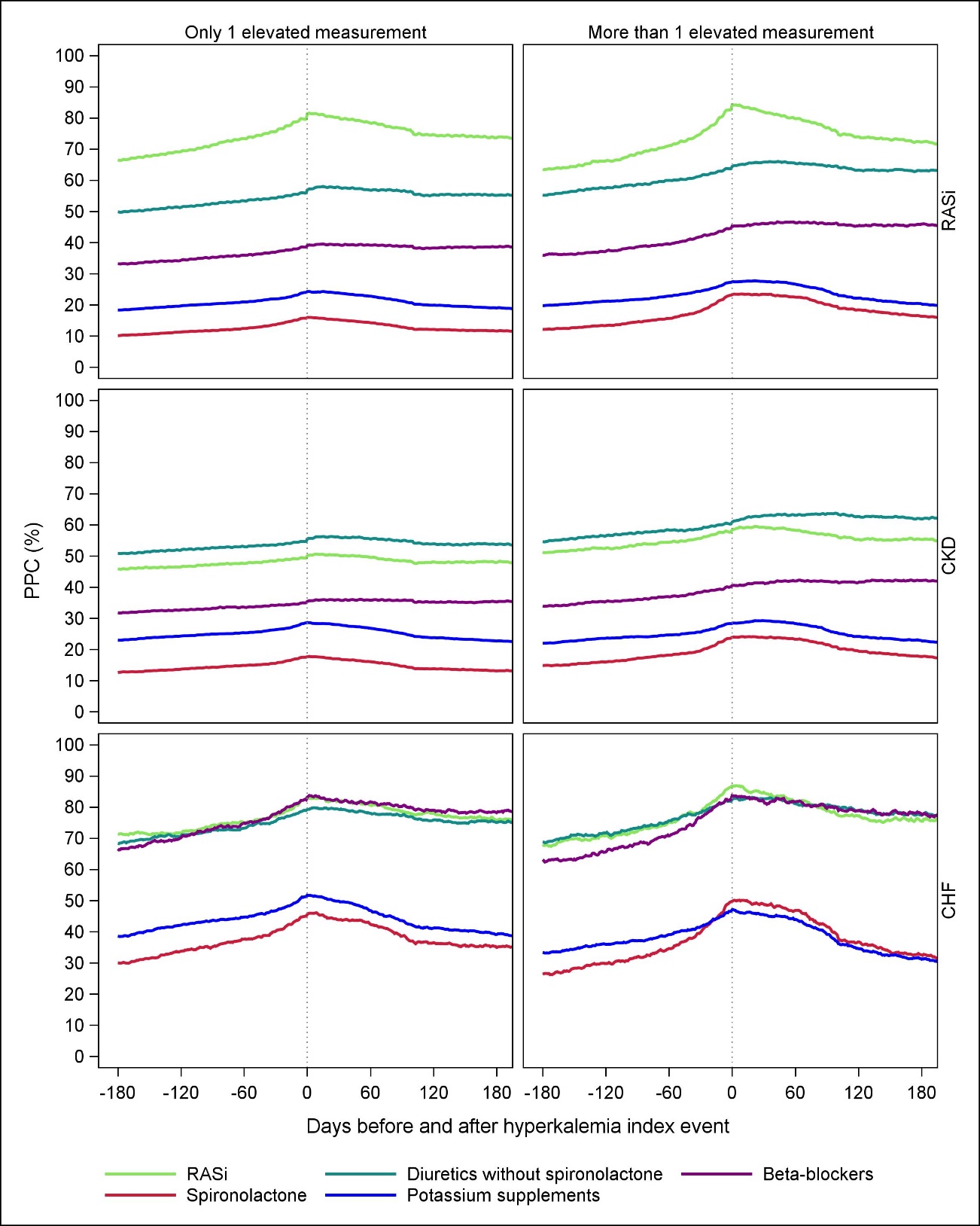


^a^Although all patients enrolled in the RASi cohort received RASi treatment at cohort entry, only 80% of the patients received treatment at the time of hyperkalemia.

^b^Due to a low number of patients receiving dialysis and in-hospital care before and after hyperkalemia, these data were omitted from the figure.

Abbreviations: CHF, chronic heart failure; CKD, chronic kidney disease; PPC, proportion of patients covered; RASi, renin angiotensin system inhibitors
